# SNV-FEAST: microbial source tracking with single nucleotide variants

**DOI:** 10.1186/s13059-023-02927-8

**Published:** 2023-04-30

**Authors:** Leah Briscoe, Eran Halperin, Nandita R. Garud

**Affiliations:** 1 Bioinformatics Interdepartmental Program, University of California Los Angeles, Los Angeles, CA USA; 2 Department of Computer Science, University of California Los Angeles, Los Angeles, CA USA; 3 Department of Human Genetics, David Geffen School of Medicine, University of California Los Angeles, Los Angeles, CA USA; 4 Department of Computational Medicine, David Geffen School of Medicine, University of California Los Angeles, Los Angeles, CA USA; 5 Department of Anesthesiology and Perioperative Medicine, David Geffen School of Medicine, University of California Los Angeles, Los Angeles, CA USA; 6 Institute of Precision Health, University of California Los Angeles, Los Angeles, CA USA; 7 Department of Ecology and Evolutionary Biology, University of California Los Angeles, Los Angeles, CA USA

**Keywords:** Source tracking, Microbiome, Single nucleotide variants, Transmission, Strains

## Abstract

Elucidating the sources of a microbiome can provide insight into the ecological dynamics responsible for the formation of these communities. Source tracking approaches to date leverage species abundance information; however, single nucleotide variants (SNVs) may be more informative because of their high specificity to certain sources. To overcome the computational burden of utilizing all SNVs for a given sample, we introduce a novel method to identify signature SNVs for source tracking. Signature SNVs used as input into a previously designed source tracking algorithm, FEAST, can more accurately estimate contributions than species and provide novel insights, demonstrated in three case studies.

**Supplementary Information:**

The online version contains supplementary material available at 10.1186/s13059-023-02927-8.

## Background

Understanding the sources that could contribute to the formation of a given microbiome is of great interest in elucidating the ecological processes that give rise to these complex communities and the impact of these communities on human and environmental health. For example, a hospital environment may introduce antibiotic resistance genes to an infant’s gut microbiome, and local selective pressures may result in vastly different microbial compositions in different parts of the ocean. Approaches for determining the proportion of a microbiome of interest (the “sink”) that is attributed to different microbiomes (the “sources”) are known as “source tracking” [1, 2]. Source tracking is useful for forensics, categorization of samples, detecting contamination, and tracing transmissions between different hosts or environments. While source tracking was developed as a way to quantitatively characterize a sample based on a set of samples with known origin, in most studies, the true source of samples may never be collected. In these cases, source tracking approaches are useful in identifying similarities between microbiome samples even if they cannot be used to definitively identify the true source of origin.

Current approaches for source tracking include the Bayesian approach, SourceTracker [1], and more recently the expectation–maximization approach, FEAST [2]. These source tracking methods use species abundance profiles of the sample of interest (the sink) and of potential sources and compute percentages of sinks that are attributable to each potential source. However, species abundance profiles miss important sub-species single nucleotide variants (SNVs), which may provide higher resolution information than species about transmission patterns. For example, Nayfach et al. [3] found that the sharing of microbiome SNVs private to mothers and their infants decreases over the first year of the infant’s life while species sharing increases. This suggests that while the infant microbiome increasingly resembles the adult microbiome ecologically, sources other than the mother also colonize the infant. Thus, species-level resolution may obscure true sources of microbes while SNVs can reveal actual transmissions to the infant.

**Fig. 1:**
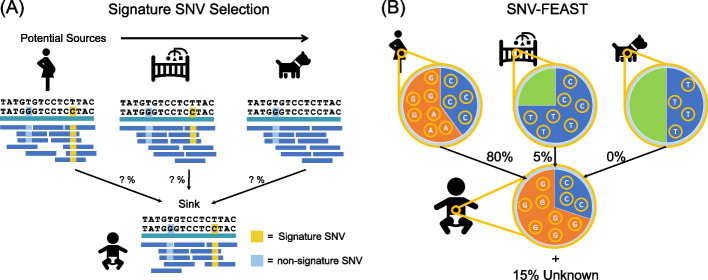
Signature SNV selection and SNV-FEAST. **A** A signature SNV is present in one or few but not all sources. By contrast, a non-signature SNV is generically present in multiple sources and thus provides little discriminating information. **B** SNV-FEAST estimates the proportion a given sink derived from various sources using the read counts for each allele in sinks and sources

While tracking strain transmissions with SNVs has been highly successful in a number of studies [3–9], current approaches to strain tracking are limited. These methods provide binary information by inferring whether or not a strain transmission has occurred per species but they do not shed light on the relative proportions of microbiomes that are similar. A specific example of this is inStrain [6] which computes a pairwise population-level average nucleotide identity (popANI) between two samples. If an infant harbors several strains derived from the mother at low frequency, these shared strains will have high popANI values, but they will represent a relatively small proportion of the infant’s microbiome. By contrast, source tracking allows us to simultaneously infer the putative proportions for multiple sources contributing to a given sink, integrated over all community members in the sink. As shown in Fig. 1, one may be able to estimate that an infant microbiome is explained 25% by their mother, 10% by their dog, and 30% by unknown sources [1, 2]. In other words, source tracking with SNVs leverages not only the genetic variants within species, but also the relative abundances of the species that carry the SNVs.

Here, we evaluate whether source contributions estimated with SNVs are more accurate than with only species when they are provided as input to FEAST [2] (hereafter referred to as SNV-FEAST and species-FEAST, respectively). FEAST [2] is faster and more accurate than previous source tracking tools [1] and therefore is ideal for adaptation to SNV source tracking since it can accept larger numbers of features and input sources. Despite this improved computational efficiency, the potentially millions of single nucleotide variants across all microbiome species in a given host still can computationally overwhelm FEAST. To address this, we introduce a novel approach to determine signature SNVs that can be used as input to FEAST. This both reduces memory requirements and computation time in the FEAST estimation, allowing us to optimally estimate the source contribution of a sink. We find that SNV-FEAST and species-FEAST yield different outcomes when applied to simulated data, with SNV-FEAST frequently out-performing species-FEAST. We apply SNV-FEAST to three real-world case studies, including source tracking between infants and their mothers in the first year of life, between infants and the neonatal intensive care unit (NICU), and between oceans around the world. We confirm the ability of SNV-FEAST by recapitulating several previously published findings in our case studies, as well as discover new source tracking patterns across oceans. In sum, we show that SNVs can be used to estimate potential transmissions across hosts and across environments.

## Results

### SNV-FEAST algorithm

Here we adapt FEAST to accept SNV abundance instead of species abundance as input. A computational challenge in using SNVs instead of species as input to FEAST is that SNVs contribute a significantly larger feature space. The number of different species comprising a microbiome can range from a few hundred to a few thousand, while the number of possible SNVs for a given species alone can be in the thousands [10]. This difference in the number of input features can result in FEAST runtimes that last several hours instead of a few minutes and memory-intensive storage of read counts at all sites of variation.

We devised a likelihood-based approach for selecting a set of informative or “signature” SNVs for a given source tracking analysis, allowing us to overcome the time and memory-intensive challenges of utilizing SNV-level data. We identify these informative SNVs by computing a signature score (Fig. 1A) (see the “Methods” section) that quantifies the extent to which SNVs in the sink are most likely derived from one of the potential sources. This is analogous to identifying SNVs private to sources and their sinks, but more generalized to include SNVs that may be found in multiple sources, albeit at higher frequency in one of the potential sources (see the “Methods” section).

To compute a signature score for a given SNV, two hypotheses are compared for each potential source: (1) that one source solely explains the observed allele counts in the sink and (2) all sources except that one source collectively explain the observed allele counts in the sink. For each hypothesis, we calculate the binomial log-likelihood for the estimate of the allele frequency in the sink, θ.

*Hypothesis 1*: Source *i* with allele frequency ${p}_{i}$ explains the allele counts in the sink.

$$\widehat{\uptheta }={p}_{i}$$

*Hypothesis 2*: A combination of all other sources except *i* (sources $j\ne$*i*) explains the observed allele count distribution in the sink. The estimate of the sink allele frequency is computed using a mixture of the allele frequencies ${p}_{j}$ from those sources. The mixing parameter ${\mathrm{\alpha }}_{j}$ is learned using Sequential Least Squares Programming with the constraint that $\sum\limits_{j\ne i}{\mathrm{\alpha }}_{j}=1$.

$$\widehat{\uptheta }=\sum\limits_{j\ne i}{\mathrm{\alpha }}_{j}{p}_{i}$$

The binomial log-likelihood is calculated as follows, where there are *n* reads with the reference allele and *m* reads with the alternative allele in the sink.

$$LL\left(\widehat{\uptheta }\right)=n\mathit{log}\widehat{\uptheta }+m\mathit{log}\left(1-\widehat{\uptheta }\right)$$

A log-likelihood ratio representing the support for hypothesis 1 relative to hypothesis 2 is calculated per site per potential source. The maximum log-likelihood ratio per site is the signature score for that SNV, representing how favorably one of the sources explains the sink over all other sources. Signature SNVs are those with scores greater than two standard deviations over the mean signature score computed for all SNVs (see the “Methods” section).

### Evaluation of SNV-FEAST in simulations

To compare the accuracy of species-FEAST and SNV-FEAST, we performed simulations mimicking mother-infant transmissions with the goal of estimating contributions of different sources to an infant sink. Our simulations tested the ability of SNVs and species to recapitulate the true source composition in synthetic samples comprised of a mixture of reads drawn from multiple real fecal adult samples. To construct these synthetic infant microbiomes, we mixed metagenomic data from mothers sampled in a mother-infant dataset [11] at various proportions as described below (see the “Methods” section).

The difficulty of source tracking increases with the number of contributing sources [2]. Thus, we simulate infants that have a small (≤ 5) versus large (6–10) number of contributing sources (Additional file 1: Table S1), including an unknown source (e.g., a randomly selected unrelated mother). Known source contributions to the simulated gut microbiome sample of the infant varied between 1 and 90% while the unknown contribution varied between 10 and 90%. The unknown source was not presented to FEAST as a potential known source.

Additionally, not all species in a mother are transmitted to the infant [5, 7, 12–14]. Thus, in our simulations, species transmission rates were determined using a beta distribution, which is a natural model for values between (0, 1) and often proposed for microbial abundance data [15–18] (see the “Methods” section). We therefore consider four simulated scenarios: a combination of low versus high number of sources and low versus high transmission rates (see the “Methods” section).

**Fig. 2:**
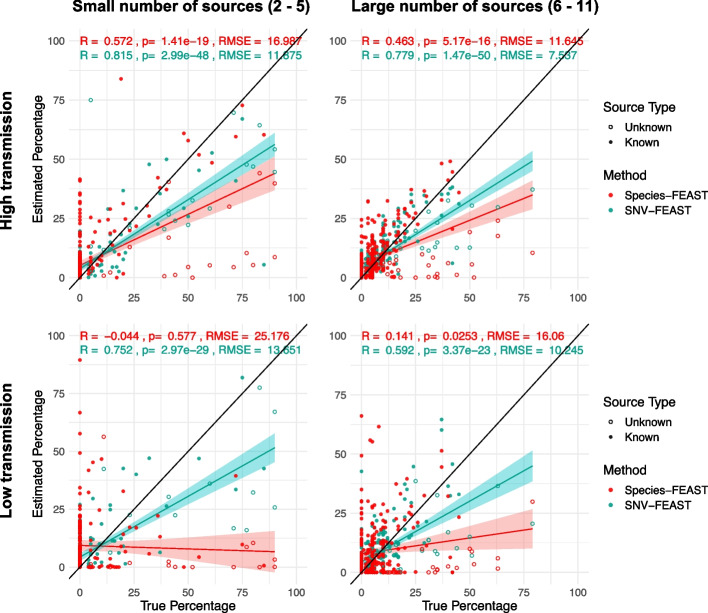
Ability of SNV and species-FEAST to recapitulate true contributions in simulations. Estimated known and unknown source proportions for infant microbiomes simulated with in silico mixtures of real maternal fecal microbiomes under different scenarios: either a small number of contributing sources (≤ 5) or large number of sources (6–11), and a high transmission rate of species or low transmission rate. The transmission rate is the probability of an infant being colonized by a given species, simulated using a beta distribution centered on the relative abundance of species in sources (see the “Methods” section). Twenty-three infants were simulated with five or fewer sources and 19 infants were simulated with a large number of sources (Table S1). The black line indicates the ground truth for proportions. For each simulated infant, there are 11 points plotted, whereby 10 correspond to known sources (some of which have zero contribution), and one corresponds to an unknown source which is indicated by hollow circles in the plot

Figure 2 compares the performance of SNV-FEAST and species-FEAST in estimating the true contribution of sources. FEAST using SNVs has equal if not better performance than species in most scenarios and performs especially well when transmission rates are low and unknown source proportions are high. SNVs have a lower root mean squared error (RMSE) compared to species in three of the four scenarios and higher Pearson correlation between true and estimated contributions in all four scenarios. The difference in these correlations for SNVs versus species is significant in all four cases when using a paired Wilcoxon signed rank test (high transmission: *p*-value = 0.00560, 0.00251 for small and large number of sources, low transmission: *p*-value = 0.00024, 0.002340 for small and large number of sources). These results suggest that SNVs may offer useful signatures of transmission.

To assess whether all species and all signatures SNVs in the sink are needed for accurate source tracking, we varied the proportion of species (10%, 50%, or 100%) and SNVs (10%, 50%, or 100%) included as inputs to the algorithm (Additional file 1: Fig. S1). We used Pearson correlation between the true and estimated proportions to represent the accuracy of SNV-FEAST. When decreasing the percentage of SNVs used, there is no statistically significant change in the performance. However, when decreasing the percentage of species used, there are statistically significant decreases in the performance (Additional file 1: Fig. S1).

To illustrate the advantage of SNV-FEAST over traditional strain tracking approaches such as inStrain [6], we used the same synthetic communities produced in the above simulation for inStrain profiling between each infant and each of their potential contributing sources (Additional file 1: Fig. S2). InStrain computes a popANI score, which represents the average nucleotide identity between two different metagenomic samples for a given species. As per the inStrain paper, popANI values > 99.999% represent the same strain being shared between samples for a given species (see the “Methods” section). However, this approach provides a binarization as to whether or not a strain was transmitted and does not account for the relative abundance of the strain in the sink. Thus, we computed the fraction of each infant’s species that have popANI $\ge$ 99.999%, with each potential source.

As expected, both SNV-FEAST and inStrain produce estimates of sharing that correlate positively with the ground truth mixture proportions of the contributing source samples in each infant (Additional file 1: Fig. S2). We found inStrain results yielded a 0.742 Pearson correlation (*p*-value < 1 × 10^−12^) with the true mixture proportions, whereas SNV-FEAST has a 0.866 Pearson correlation (*p*-value < 1 × 10^−12^) with the true proportions. The higher correlation values for SNV-FEAST likely reflect that relative abundances of strains and their genomic identities are simultaneously taken into account for source tracking, whereas inStrain only accounts for genomic identities. Finally, several of the shared species in the simulations had popANI values < 99.999%, reflecting the complex mixtures from multiple sources.

We next compared SNV-FEAST with the strain tracking procedure in Nayfach et al. [3]. Again, we used the same synthetic communities produced in the simulation to determine marker alleles as defined in Nayfach et al. [3] (see the “Methods” section). Here a marker allele is determined to be a SNV that is private to mother, infant, or the mother-infant dyad, and absent from the background population, which consisted of other samples in the dataset as well as samples from US adults in the Human Microbiome Project [19, 20] (see the “Methods” section). Species with $\ge$ 5% marker allele sharing between mother and infant were deemed to share a strain (see the “Methods” section). We found a high correlation between the true mixture proportions (on *x*-axis) and the percentage of species with transmission events (*y*-axis) (Pearson correlation 0.915, *p*-value < 1 × 10^−16^) (Additional file 1: Fig. S3A). The higher correlation for the Nayfach et al. [3] approach compared to the inStrain approach possibly reflects horizontal gene transfers between lineages residing in infants and mothers. By contrast, there was a lower correlation between the true mixture proportions (*x*-axis) and the sharing for all marker alleles across species present in the infant (*y*-axis) and (0.575 Pearson correlation, *p*-value < 1 × 10^−16^) (Additional file 1: Fig. S3B).

### Source tracking in infants over the first year of life

Having assessed the abilities of SNV-FEAST in synthetic data, we next estimated the contribution from the true mother over time to the true infant with SNV and species-FEAST in the Bäckhed et al. [11] dataset. This dataset is composed of metagenomic samples from infants collected at 4 days, 4 months, and 12 months after birth, as well as their mothers at the time of delivery. Previous analyses on this data have shown that even while species similarity increases, infants and their mothers share fewer proportions of strains over time as revealed by sharing of SNVs private to mother-infant dyads [3]. Thus, SNVs belonging to strains shared only by the infant and their mother may be more informative of the true source compared to species. Here we sought to test whether SNV and species-FEAST recapitulate these results (see the “Methods” section).

In applying FEAST to the Bäckhed et al. [11] dataset, we estimated the proportion of the infant sample at birth attributable to their own mother. For 4-month-old infants, we estimated the proportion attributable to the mother and itself at birth. For 12-month-old infants, we estimated the proportion attributable to the mother and itself at birth and 4 months [2]. This allowed “unknown” to be more strictly defined as the component of the infant microbiome that could not be explained by the mother. It also allowed us to better discern if completely new strains were acquired at the 4th and 12th months of life (that were not already acquired during previous life stages).

**Fig. 3:**
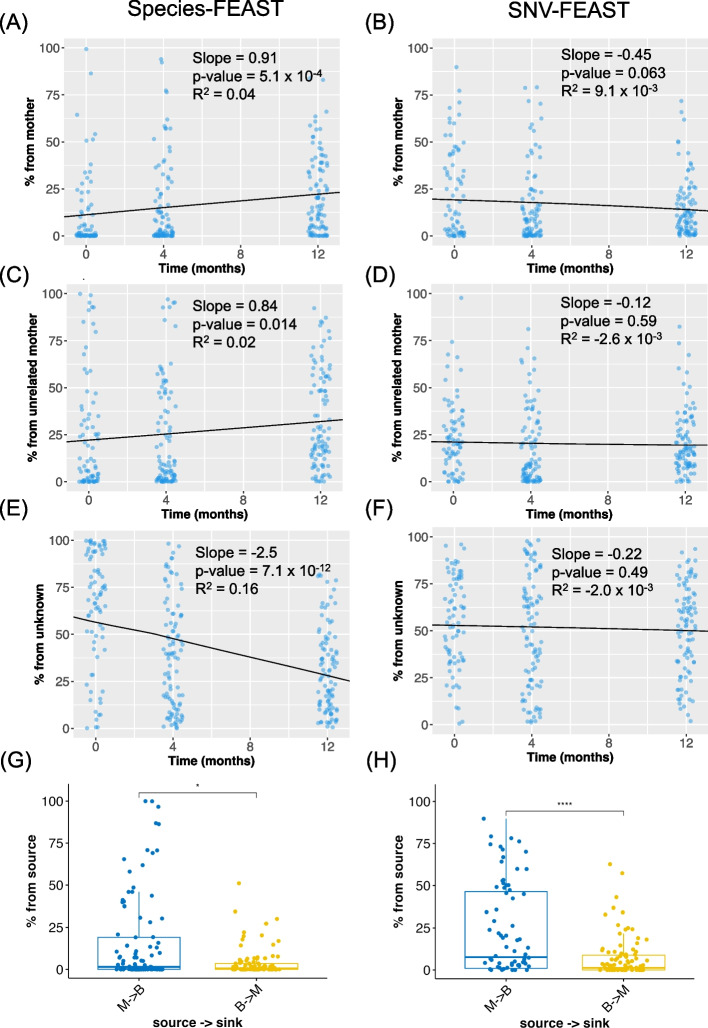
Source tracking in the infant gut microbiome over the first year of life. Species- and SNV-FEAST were applied to Backhed et al. 2019 data to estimate the contribution of **A**, **B** mother, **C**, **D** unrelated mothers, and **E**, **F** unknown sources to infants sampled at birth, 4 months, and 12 months. The black line and inset statistics pertain to the linear regression fit for the source estimates as a function of age of the infant. **G**, **H** are swapped source tracking analyses with mother and infant swapped when using species-FEAST and SNV-FEAST, respectively. Additional file 1: Fig. S4 shows the species that were included in species-FEAST and species that had SNVs included in SNV-FEAST. Additional file 1: Fig. S5 shows the estimate of the unknown component when previous time points of the infant are excluded from the sources

First, consistent with previous findings made with species and SNVs [3], species-FEAST estimates an increasing contribution of the mother over time (*t*-test *p*-value = 5.1 × 10^−4^), but SNV-FEAST estimates a decrease over time (*p*-value = 0.063) (Fig. 3).

Second, we assessed the ability of species and SNV-FEAST to distinguish the true mother from three randomly selected unrelated mothers. Species-FEAST estimates an increasing contribution of unrelated mothers over time (*t*-test *p*-value = 0.014) while SNV-FEAST estimates no significant change over time (*t*-test *p*-value = 0.59) (Fig. 3). The increase in contribution from unrelated mothers with species-FEAST does not suggest that these particular unrelated mothers are seeding the infant. Rather, the opposing trend observed with SNVs suggests that similarity at the species level is consistent with the maturation of the infant microbiome over time.

Finally, we estimated contributions from unknown sources, i.e., the proportion of the infant microbiome not explainable by the true mother, the three randomly selected unrelated mothers, or any previous time point. Species-FEAST estimates a sharp decline in the contribution of unknown sources over the first year of life (*t*-test *p*-value = 7.1 × 10^−12^) (Fig. 3). This significant decrease in unknown at the species level reflects the infant microbiome maturation over the first year of life. By contrast, SNV-FEAST estimates little change in the contribution of unknown sources (*t*-test *p*-value = 0.49) (Fig. 3). Note that this unknown component reflects what was gained since a previous time point. In other words, at 12 months, the infant on average acquired the same fraction of unknown as it did at 4 months and birth. When source tracking is run without including previous time points as sources, the unknown component increases over the first year of life for SNVs only (Additional file 1: Fig. S5).

Next, we sought to understand the effect of swapping sink and source in the re-analysis of Bäckhed et al. [11] data. In Fig. 3G and H, the infant at birth is the potential source and the mother is the sink. The estimated contribution from baby to mother is significantly smaller (species-FEAST: 11.9 difference, Wilcoxon rank sum test *p*-value = 0.013; SNV-FEAST: 16.0 difference, *p*-value = 2.2 × 10^−5^) compared to that of mother to baby. This trend may be suggestive, but is not conclusive, of directionality, whereby a less diverse source is seeded by a more diverse source.

### Contribution of the NICU-built environment to infant microbiomes

Next, we re-analyzed a metagenomic dataset studying the contribution of the hospital environment to the infant gut microbiome in the neonatal intensive care unit (NICU) [21]. This dataset is composed of microbiomes of infant stool, as well as the NICU rooms of the same infants at frequently touched surfaces, sink basins, the floor, and isolette-top sampled over an 11-month period [21]. We applied SNV and species-FEAST to assess the contribution of the infant’s own NICU room as well as a different NICU room in the vicinity to the infant’s gut microbiome (see the “Methods” section).

**Fig. 4:**
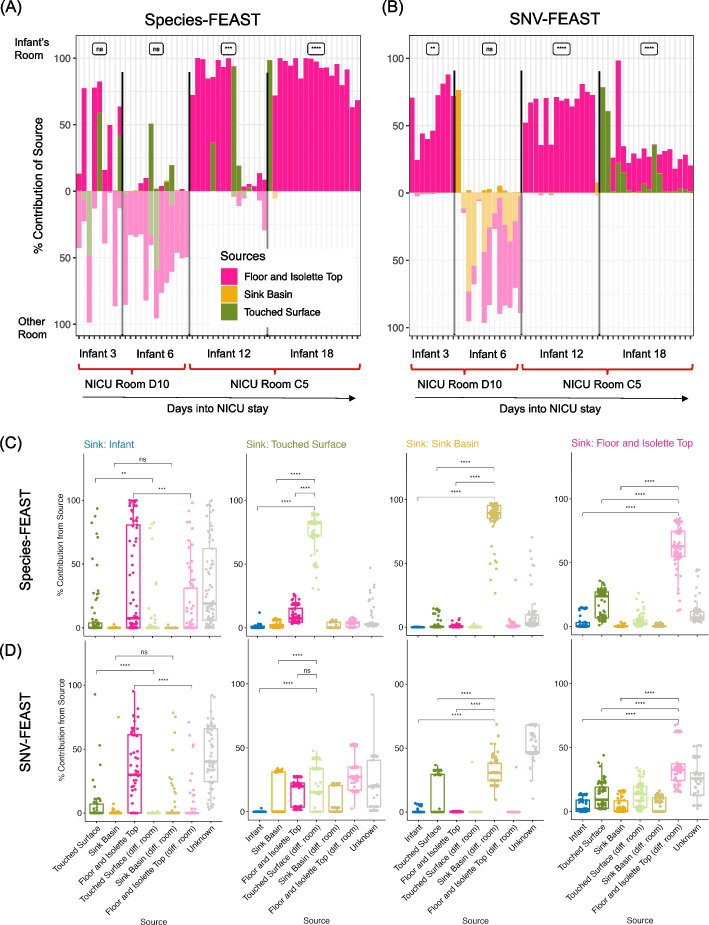
Source tracking of infant gut microbiome in the NICU. **A** Species-FEAST and **B** SNV-FEAST applied to infants in the NICU. Each bar represents one sampling day in the NICU stay of an infant. Infants 3 and 6 stayed in the same room, but at different times. The same applies to Infants 12 and 18. The contribution of a different room was determined by using samples from Infant 12’s room for Infants 3 and 6, and samples from Infants 6’s room for Infants 12 and 18 for each of the categories of surfaces per infant: touched surface, sink basin, or floor and isolette top surface. The asterisks represent the result of a paired Wilcoxon signed rank test indicating whether the total contribution of surfaces from the infant’s own room was higher than contributions from the other room. Iterative swapping of the infant sink and each potential source for source tracking with **C** species-FEAST and **D** SNV-FEAST. The first column shows source tracking results in which the infant was treated as the sink. In each column after the first column, a different environmental source was swapped with the infant and treated as a sink. The brackets indicate the pairs of results that are compared using a paired Wilcoxon signed rank test. For all results, the following symbols represent the results of the statistical test: **** for *p*-value < 0.0001, *** for *p*-value < 0.001, ** for* p*-value < 0.001, * for* p*-value < 0.05, and n.s. for *p*-value > 0.05

Concordant with the findings of Brooks et al., both SNV and species-FEAST detected that the most common source contributing to the infant microbiome was the floor and isolette-top from the infant’s own room (Fig. 4A, B). SNV-FEAST found Infant 18 also had large contributions from their own room’s touched surfaces at multiple time points (Fig. 4B), which is consistent with a finding by Brooks et al. that three strains found in Infant 18 perfectly matched (> 99.999% average nucleotide identity) strains found in the touched surfaces samples of Infant 18’s own room. Lastly, both species-FEAST and SNV-FEAST found Infant 6’s microbiome was explained almost entirely by samples from a different room with SNV-FEAST finding a sizeable contribution from both the floor and isolette top and the sink basin in this different room. This is concordant with Brooks et al.’s finding of multiple cases of strain sharing across rooms of Infant 6 and 12 for the different surfaces. FEAST with both data types can quantify the extent to which Infant 6’s microbiome was influenced by strains present in the built environment.

Through application of SNV and species-FEAST, we can quantify any time trends for the influence of the built environment on the infant microbiome (Fig. 4A, B). SNV-FEAST more consistently finds that contribution from the infant’s own room exceeds contributions from a different room over time (paired Wilcoxon signed rank test for same room > different room: Infant 3: *p*-value = 1.95 × 10^−9^, Infant 6: 1.0, Infant 12: 3.05 × 10^−5^, Infant 18: 3.81 × 10^−6^) as compared to species-FEAST (Infant 3: *p*-value = 0.41, Infant 6: 1.0, Infant 12: 5.8 × 10^−4^, Infant 18: 3.81 × 10^−6^). Interestingly, species-FEAST assigns one dominant source primarily, whereas SNV-FEAST more often finds a combination of sources for a given sample.

Additionally, both SNV and species-FEAST estimated a large unknown component for all four infants, with Infant 18 showing the largest mean unknown component across the NICU stay based on SNVs (Additional file 1: Fig. S6). This unknown component is important because it signifies the extent to which other sources such as the mother and diet impact infant gut colonization.

We then asked the question is the infant more explained by the built environment rather than vice-versa, the built environment is more explained by the infant. We tested this by swapping the infant and each of the three built environment sources (Fig. 4C, D). The estimated contribution of room to infant is significantly higher than the estimated contribution of infant to room, but this asymmetry is more pronounced with SNV-FEAST. SNV-FEAST showed significantly higher contribution of room to infant for two of the three surface types (floor and isolette top: Wilcoxon rank sum test *p*-value = 7.00 × 10^−9^, touched surface: *p*-value = 0.0058, sink basin: *p*-value = 0.274) while species-FEAST found this to be true for one of the three surface types (floor and isolette top: Wilcoxon rank sum test *p*-value = 7.1 × 10^−5^, touched surface: *p*-value = 0.968, sink basin: *p*-value = 0.998). Interestingly, the built environments of different rooms highly resemble each other. This is especially apparent with species-FEAST, suggestive of similar ecological forces operating in similar built environments. By contrast, SNV-FEAST reveals a higher diversity of contributing sources of the built environment samples to other NICU-built environments, once again highlighting the utility of performing source tracking with SNVs.

### Global source tracking of ocean microbiomes

The ocean microbiome is a complex community that displays biogeography at the species and functional levels [3, 22]. To further understand global patterns of ocean microbiomes, we applied SNV and species-FEAST to the Tara Oceans microbiome dataset [22]. In the source tracking context, rather than defining sharing as evidence of a transmission event (which is more likely in mother-infant data), estimated source contributions at best explain the extent to which a given ocean sample resembles other ocean samples. On one extreme, an ocean sample might be entirely explainable by a single ocean’s samples, and at the other extreme, an ocean sample might be explainable by multiple oceans at the same time. Another alternative is for an ocean sample to not be explainable by any of the provided sources, resulting in a high unknown component and potentially suggesting high endemism. These source tracking estimates could be indicative of the extent to which oceans mix or may be reflective of similar niches.

Tara Oceans is composed of 182 whole metagenomic sequencing samples derived from 64 stations at multiple depths. Previous research indicates that temperature is one of the highest drivers of variability in microbial composition in the ocean [22, 23]. For this reason, we restricted the source tracking analysis to sinks and sources from the same temperature and depth range: above 20 °C and within an average of 5 m below the surface.

**Fig. 5:**
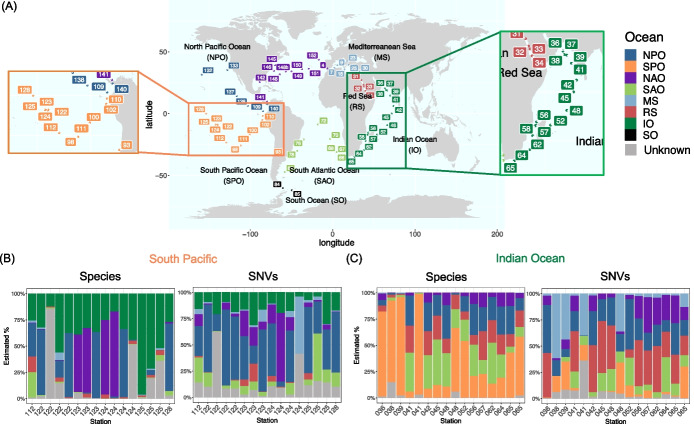
Microbial source tracking in the Tara Oceans dataset with SNV and species-FEAST. **A** World map indicating the location of sampling sites. Source tracking estimates for the contribution of different oceans to the **B** South Pacific (*n* = 16) and **C** Indian Oceans (*n* = 16) are depicted with vertical bars. In each experiment, all stations around the world excluding those from the “sink” ocean are treated as potential sources. Light blue, for example, represents the total contribution of the four stations from the Mediterranean Sea that had samples in the surface layer that were also greater than 20 °C in temperature

First, we performed source tracking between oceans using SNV and species-FEAST. We treated each station around the world as a sink and estimated the contribution of different oceans around the world to that sink (see the “Methods” section). Unknown represents any portion of the microbiome in these sink samples that cannot be explained by any of the provided source samples. We found that species and SNV-FEAST estimate different amounts of sharing between oceans, where SNVs estimate a higher unknown on average, potentially indicative of endemism. The finding that SNV-FEAST estimates a higher unknown contribution on average is most evident in the North Pacific, North Atlantic, South Atlantic, and Mediterranean oceans (Additional file 1: Fig. S7). Additionally, in some oceans, SNVs identify contributions from oceans that species-FEAST does not detect (Fig. 5, Additional file 1: Fig. S7). For example, in applying FEAST to Indian Ocean samples, we find that there is measurable sharing of microbes with the Mediterranean Sea, but this is not detected with species (Fig. 5C). Such differences were found in samples from other oceans as well (Additional file 1: Fig. S7).

**Fig. 6:**
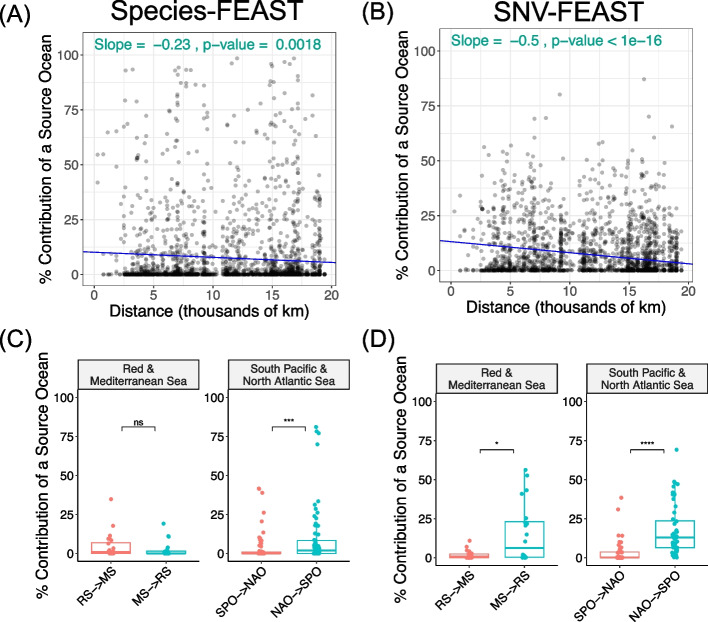
Source tracking with ocean samples. Distance decay in the contribution of a “source” ocean to a “sink” ocean when using **A** species-FEAST and **B** SNV-FEAST. In each experiment, only stations from one ocean were considered sources for a given sink station. For example, when performing source tracking between the Mediterranean and North Atlantic, for each Mediterranean station, the 10 available North Atlantic stations were considered potential sources. Thus, plotted are 10 points for a given Mediterranean sink, where each point represents the contribution of a source station from the North Atlantic to the Mediterranean sink station in question. Shown in the inset text are the slope and *t*-test *p*-value for the slope. **C** and **D** are flipped source tracking analysis with the Red Sea and Mediterranean, as well as the South Pacific Ocean and North Atlantic Ocean using species-FEAST and SNV-FEAST, respectively

Next, we assessed whether source tracking estimates display a distance-decay relationship. Previous studies found that genetic distance, such as that represented by fixation index F_ST_, increases with geographic distance between populations [24, 25]. Based on these findings, our expectation was that samples that are further away from a given station will have reduced resemblance to that station. To assess this distance-decay relationship, we plotted pairwise source tracking results across all possible pairs of ocean samples (Fig. 6A, B). We found that indeed as the distance increases, the % explainability of a given source ocean decreases − 0.23% per thousand km according to species-FEAST (*t*-test *p*-value < 1 × 10^–16^), and − 0.5% per thousand km according to SNV-FEAST (*t*-test *p*-value = 0.0018). The steeper slope for SNV-FEAST suggests that SNVs may be more sensitive to distance decay signals on a global level.

Finally, we investigated whether some oceans have higher estimated contributions to other oceans than vice versa, potentially indicative of the directionality of transmissions (though see the “Discussion” section). Specifically, we investigated the relationship between the Red Sea to the Mediterranean Sea (Fig. 6C, D). Migration from the Red Sea to the Mediterranean, known as Lessepsian migration, is well-documented for not only microorganisms but also macroorganisms like fish [26–28]. Additionally, recent studies may suggest that anti-Lessepsian migration of bacteria (Mediterranean to the Red Sea) is more common than Lessepsian migration [29]. Studies find that the Mediterranean has brine pools that produce a similar environment to the Red Sea’s [30], allowing for bacteria from the MS to potentially thrive in the RS.

By swapping the Red Sea and Mediterranean as source and sink, we found that there was indeed a significant difference in the estimated contribution from one direction to another with SNVs but not species (Fig. 6C, D). SNV-FEAST found the Mediterranean explained an average of 15% of the Red Sea, while the Red Sea explained an average of 1.8% of the Mediterranean (Wilcoxon rank sum test, *p*-value = 0.02), consistent with anti-Lessepsian migration. Meanwhile, a similar analysis with species-FEAST found the Mediterranean explained 2.5% of the Red Sea and the Red Sea explained 4.9% of the Mediterranean (Wilcoxon rank sum test, *p*-value = 0.25). In a similar analysis between North Atlantic and South Pacific, we found that both species and SNVs supported significantly greater contributions from the North Atlantic to the South Pacific, with SNV-FEAST estimating a greater contribution (17%, Wilcoxon rank sum test *p*-value = 5.1 × 10^−11^) than species-FEAST (10%, Wilcoxon rank sum test p-value = 1.8 × 10^−4^). The same analysis performed in the other oceans is presented in Additional file 1: Fig. S8.

Together, these results suggest that on average, SNV and species FEAST generate similar source tracking results in the Tara Oceans dataset, with SNVs displaying stronger signals of endemism, distance-decay relationships, and potential directionality of transmission.

## Discussion

Source tracking provides insight into potential source contributions to a metagenomic sample as well as similarities between metagenomic samples. While species abundances have been informative in source tracking in several studies [1, 2, 31–33], they may be limited in their resolution. SNVs provide a potential alternative because of their ability to distinguish sources of strain transmissions. Here we compared the ability of a previously published source tracking algorithm FEAST using species versus SNVs as input data. In the application of species and SNV-FEAST to simulations as well as three case studies, we demonstrate that the two input types can provide distinct insights into microbial sharing and similarities across different environments. As a hypothetical example, two unrelated samples may have very similar species composition due to similar colonization processes and similar environmental influences without any actual microbial sharing. It would be unlikely for these two unrelated samples to share rare SNVs, however. This distinction suggests that SNVs indeed can provide insight into the ecological processes shaping microbial communities that species information alone cannot, and our three case studies are able to demonstrate this.

In the first case study, we confirmed previous findings that SNV sharing between mothers and infants decreases over the first year of life while species sharing increases [3], suggesting that while the infant microbiome matures to resemble adults at the species level, sources other than the mother may seed the infant over time. In the second case study, we confirmed source contributions from the NICU-built environment to the infant microbiome [21] and found that SNVs detect a more consistent estimate in source contributions over time compared to species as well as detecting contribution from sources not detectable by species-FEAST.

In the third case study, we perform source tracking in the Tara oceans dataset [22] and found SNVs display a stronger distance decay relationship than species. These distance-decay results parallel recent findings made with gene content [34]. While previous studies have examined the biogeography of the ocean using species profiles, genes [3, 34], or amino acid variants from a single species (SAR11) [35], for the first time, we leverage the use of SNVs across all detected prevalent species in the ocean microbiome to identify proportions of sharing across oceans. A benefit of using SNVs in the ocean microbiome is that SNVs can track fragments of DNA that have moved due to horizontal gene transfer in the distant past rather than relying on inference of whole genomes or presence of private SNVs that may be transmitted in the recent past. This global-level source tracking is analogous to admixture estimation with human genotypes [36, 37].

We note that source tracking provides insights into similarities between microbiomes and potential transmissions, though the directionality is less conclusive. It is possible that increased contributions in one direction but not the other are suggestive of the directionality of transmission. For example, in the case of the mother-infant data from Bäckhed et al. [11], FEAST predicted a higher contribution from mother to baby than vice versa. This is consistent with work done on crAss-like phage transmissions between mother and infant in the same dataset that showed evidence of directionality by tracking the accumulation of mutations over time that are private to the infant and absent from the mother [38]. But in the case of the ocean, it is possible that over longer time periods, differences in relative contributions from one part of the world to another (e.g., Mediterranean to Red Sea) are more reflective of local selection pressures that permit certain species and genotypes [35]. Thus, source tracking in certain instances, such as the ocean microbiome, at best reflects the extent of similarity between samples and is less conclusive about directionality.

A popular approach used to track strain transmissions is by detecting high average nucleotide identity (ANI) for species shared between source and sink. For example, inStrain [6] identifies a match between samples for a given species when ANI exceeds 99.999%. However, it is to be noted that inStrain provides distinct and complementary information from FEAST given its binarization of whether or not a strain is shared. For illustration purposes, if an infant harbors 100 species, of which only 1 came from their mother, but that species’ strain’s relative abundance is 50% of the infant’s microbiome, SNV-FEAST would infer that the mother’s contribution is 50%, while inStrain would infer that only 1/100th of the infant’s species are derived from the mother.

Other studies rely on tracking transmissions of strains with private SNVs shared only between the sink and putative source [3, 7, 9, 11]. The private marker allele tracking approach in Nayfach et al. [3] provides an improved estimate of true percentage of species that share some portion of their genome with putative sources compared to inStrain (Additional file 1: Fig. S2, S3). It is possible that requiring only 5% of marker alleles to be shared rather than a 99.999% ANI permits the detection of horizontal gene transfers between lineages residing in mothers and infants [39, 40]. However, in FEAST, by using any SNV with an informative distribution across sources as determined by our signature scoring method, we are able to quantify the relative contribution of all the sampled environments and assign a proportion to these putative sources. Another advantage to FEAST is that the contribution of unknown sources can be quantified. For example, the significant fraction of marine biodiversity estimated to be “unknown” may be endemic, as previously noted in the Mediterranean [41].

A drawback, however, with using SNVs over species is deeper, whole genome sequencing is required to accurately call SNVs. Moreover, even when there is sufficient coverage, there is still the challenge of a large number of SNVs that make FEAST computationally prohibitive. We demonstrate one way to subset SNVs that uses a scoring method for informativeness, but there may yet be other methods for filtering SNVs to the most informative set. Another potential caveat of SNV filtering is that not all species present will be represented in the final signature SNV set (Additional file 1: Fig. S4). Species with higher abundance are more likely to be represented in the signature SNV set. However, we show that not all species need to contribute signature SNVs in order to make accurate inferences, and likewise, not all SNVs are needed to make accurate inferences (Additional file 1: Fig. S1).

Ascertainment of SNVs from metagenomic data in a high-throughput manner, especially common SNVs with microbiome genotyping technology [42], is becoming an increasing priority for the field as metagenomic datasets become more abundant. A genotyper for prokaryotes has already been developed and tested on a catalog of over 100 million SNVs in order to characterize population structure [42]. Such a catalog of informative SNVs could be invaluable for source tracking. With source tracking enabling us to characterize samples by their relationship to known samples, we have a powerful tool to explore samples in new contexts we have yet to discover.

## Conclusions

SNV-FEAST is a novel approach to accurately perform source tracking using metagenomic data. By using our algorithm for determining signature SNVs, one can identify relevant SNVs that can be directly provided to FEAST, an existing source tracking approach that can successfully estimate sources using species abundance data. We demonstrate that SNV-FEAST not only accurately quantifies ground truth proportions in simulations but can also recapitulate previous findings in real-world infant datasets. In each test scenario, SNV-FEAST and species-FEAST yield different outcomes, with SNV-FEAST frequently out-performing species-FEAST. Finally, in applying SNV-FEAST to ocean metagenomic data, we uncovered distance-decay relationships between putative sources and sinks. With low computational cost, SNV-FEAST is able to leverage the increasing availability of shotgun metagenomic data to ask fascinating questions about microbiomes in the environment and hosts.

## Methods

### Data

For simulations and analyses of infant microbiomes in the first year of life, we downloaded the raw shotgun metagenomic sequencing reads from public read archives under accession number PRJEB6456 [11]. We downloaded the raw sequence reads for the NICU analysis from accession number PRJEB323631 [21], and the equivalent for the Tara Oceans analyses was downloaded from accession number PRJEB402 [22]. Data from the HMP Consortium [43] and Lloyd-Price et al. [20] was downloaded from the following URL: https://aws.amazon.com/datasets/human-microbiome-project/ [19].

### Estimation of species and SNV content of metagenomic samples

We used MIDAS (Metagenomic Intra-Species Diversity Analysis System), version 1.2, downloaded on November 21, 2016 [3], to estimate species abundance and SNV content per species in each metagenomic shotgun sequencing sample. The database we used to apply MIDAS consisted of 31,007 bacterial genomes that are clustered into 5952 species. The parameters we used to estimate species abundances and SNVs were described in [44]. A species was considered present if there are at least 3 reads mapping to a set of single-copy marker genes on average. To call SNVs, we used the default MIDAS settings in order to map reads to a single representative reference genome. The mapping was done with Bowtie 2 [45]: global alignment, MAPID ≥ 94.0%, READQ ≥ 20, ALN_COV ≥ 0.75, and MAPQ ≥ 20, where species with reads mapped to less than 40% of the genome were excluded from the SNV calls. We excluded samples with depth lower than 5 reads, and excluded genetic sites using the default site filters of MIDAS (e.g. ALLELE_FREQ ≥ 0.01, with the exception of SITE_DEPTH which was set to 3.

### Application of FEAST algorithm

FEAST, originally introduced by Shenhav et al. [2], is an R-based method that models the mixture proportions for various “source” microbial samples for a given “sink” [2]. This method utilizes expectation maximization to estimate the proportions when given any sort of count-based feature matrix representing the potential sources and sinks. The intuition behind the estimation process is that a source with a similar species distribution to the sink would have a higher contribution estimate to the sink. A species with non-zero counts only in source *j* and the sink would increase the estimated contribution of source* j*. However, in many cases, the same species are found in multiple sources simultaneously. The algorithm does not uniquely assign a species to a source but rather simultaneously utilizes all species information to infer the source contributions. The method was originally tested and evaluated on species and not on more fine scale genetic data such as SNVs. The number of different species, on average, ranges in number from a few hundred to a few thousand, while the number of possible nucleotide sites that vary across different sources can number in millions. For this reason, a SNV-filtering process is necessary so that the algorithm can run within a reasonable time and with reasonable memory requirements.

### Application of FEAST to the Bäckhed et al. [11] dataset

For both species and SNV-FEAST, the same set of sources and sinks were fed into the FEAST algorithm. In the case study of infants in the first year of life [11], the sink consisted of the infant fecal sample at either 4 days, 4 months, or 12 months and the potential sources consisted of fecal samples from the true mother, three randomly selected mothers from the same dataset, and also any previous time points for the infant.

Species-FEAST utilized all species present in the infant whereas SNV-FEAST used signature SNVs from the subset of species that had signature SNVs. Shown in Additional file 1: Fig. S4 is the distribution of species included in species and SNV-FEAST.

### Application of FEAST to the Brooks et al. [21] dataset

For the case study of infants in the NICU [21], the sink consisted of the fecal sample of the infant at a given time point and the potential sources consisted of pooled reads from the touched surfaces, the sink basin and the floor and isolette top from both the infant’s own room as well as a different room. The different room was Infant 12’s room for Infants 3 and 6, Infants 6’s room for Infants 12 and 18.

### Application of FEAST to the Sunagawa et al. [22] dataset

For the Tara Ocean [22] samples, the sink consisted of the surface water sample from the ocean station of interest while the sources consisted of surface water samples from every other station from every other ocean in the world. To study the relationship between source tracking estimates and geographic distance, we analyzed all oceans as either a sink or source against all other possible oceans. To compute geographic distance between stations, we applied the Haversine distance to the longitude and latitude of the sampling sites provided by [22] using the package “geosphere” [46]. Source tracking estimates were computed as described above using either SNV-FEAST or Species FEAST. The regression line for the distance decay analysis was computed using a linear mixed model “contribution ~ distance + (1| sink_ocean)”.

### Determining the signature SNV set

Signature SNVs were identified as described in the main text. We provide specific steps for determining signature SNVs:

1. Filter sites: only sites of the genome with at least the required number of reads mapping to the site are considered. In the case study of infants in the first year of life [11] and infants in the NICU [21], the minimum coverage requirement is 10 across the sink and *J* sources. For the Tara Ocean [22] samples, the minimum coverage is five reads [22]. Additionally, sites that are biallelic must have more than one read mapped to each allele to be considered.
2. Perform per site per source parameter estimates: for each potential source compute the estimated allele frequency in the sink θ under two different hypotheses:

*Hypothesis 1*: Source *i* with allele frequency ${p}_{i}$ explains the allele counts in the sink.

$$\widehat{\uptheta }={p}_{i}$$

*Hypothesis 2*: A combination of all other sources except *i* (sources $j\ne$*i*) explains the observed allele count distribution in the sink. The estimate of the sink allele frequency is computed using a mixture of the allele frequencies ${p}_{j}$ from those sources. The mixing parameter ${\mathrm{\alpha }}_{j}$ is learned using Sequential Least Squares Programming (scipy.minimize()) with the constraint of summing to 1 with bounds of 0 to 1 inclusive:${\sum }_{j\ne i}{\mathrm{\alpha }}_{j}=1$.

$$\widehat{\uptheta }=\sum\limits_{j\ne i}{\mathrm{\alpha }}_{j}{p}_{i}$$

(3) Compute per site per source log-likelihoods: Compute the binomial log-likelihood under hypotheses 1 and 2, given *n* reads with the reference allele and *m* reads with the alternative allele in the sink:
  $$l\left(\widehat{\uptheta }\right)=n\mathit{log}\widehat{\uptheta }+m\mathit{log}\left(1-\widehat{\uptheta }\right)$$
(4) Compute per site per source log-likelihood ratio:
  $${l}_{1}\left(\theta \right)-{l}_{2}\left(\theta \right)$$
(5) Compute per site summary signature score: The maximum log-likelihood ratio per site is the signature score for that SNV, representing how favorably one of the sources explains the sink over all other sources
(6) Filtering of SNVs using signature score: One signature score for that SNV represents how favorably one source explains the sink better than all other sources. All the scores are ranked across SNVs and SNVs with scores that are greater than two standard deviations over the mean signature score within each 200-kbp window of the genome are retained as signature SNVs. This window size was chosen for to optimize run time and memory requirements.

Note, if only one source passes minimum coverage filtering, ${l}_{2}\left(\theta \right)=0$ resulting in a very high likelihood ratio as represented by ${l}_{1}\left(\theta \right)$ for the one source. These SNVs are more likely to pass the signature score filtering. One exception for SNVs that are included in the signature SNV set without passing signature score filtering are SNVs with an allele that is completely unique to the infant, as these represent SNVs that are potentially derived from an unknown source. Signature SNVs are obtained from the SNV profile of every species for which there is MIDAS output.

### Simulating mother-to-infant transmission

The mixture proportions for 28 simulated infants are shown in Table S1. Four possible scenarios are simulated using a combination of either low or high number of sources and low or high transmission probabilities of species. High transmission of species was simulated by drawing separate transmission probabilities for each species in each contributing source based on a beta distribution with a mean equal to the species relative abundance and variance equal to 0.1, a value selected to emulate Backhed et al.’s mean relative abundance and variance. For the low transmission scenario, transmission probabilities were drawn from a beta distribution with mean 0.1 times the relative abundance of that species in the source sample and variance at 0.1. To determine if a species from each source was transmitted to a given infant, a binomial draw was performed *J* times, where *J* = number of sources, and the probability of a mother transmitting the species is *p*_*j*_ based on the beta-drawn transmission probability. If any of the draws yields value 1, that species is transmitted to the infant from all sources. The same simulated data under these scenarios is used for both SNV and species source tracking.

The source tracking estimates are compared to the true mixing proportions using Spearman correlation. The significance of correlation is calculated using the stat_cor function in the “ggpubr” package [47].

### Comparison to inStrain

We ran inStrain [6] on the same synthetic samples as described above. InStrain “profile” [6] and inStrain “compare” [6] were run for every possible infant-source pair. For example, for simulated infant 1, there were 10 putative sources; therefore, inStrain compare was run 10 times for each putative source. InStrain reports popANI calculated per scaffold for a given species. To compute a single statistic per species, we computed the average popANI across scaffolds for a given species. The percent infant microbiome species that had strains shared with mother was computed as the number of species in which popANI was ≥ 99.999% divided by the total number of species with coverage ≥ 5. PopANI was only calculated in scaffolds that had ≥ 5 coverage in both samples of the pair.

### Comparison with strain tracking approach in Nayfach et al. [3]

We applied the strain tracking approach in Nayfach et al. [3] on the same synthetic communities described above. In Nayfach et al. [3], strain transmissions are tracked by identifying “marker alleles” which are private to the infant, mother, or infant-mother dyad, and absent from the broader population. A strain is considered to be shared if at least 5% of all marker alleles for a mother-infant dyad are shared. Note that the approach for strain tracking proposed in Nayfach et al. [3] utilizes SNV information outputted by MIDAS, but is not a part of MIDAS.

Each simulated infant had up to 10 sources that were real maternal samples from Bäckhed et al. [11]. For each possible pair of infants and maternal sources (10 pairings per infant, with 48 infants), we found the set of infant-only marker alleles, mother-only marker alleles, and mother-infant dyad marker alleles. As described in Nayfach et al. [3], only sites with minimum of 30 reads and only alleles that were supported by at least 10% of the total reads aligned to that site were considered. The infant marker allele and mother marker allele were defined as alleles that were present only in the focal sample and absent from the background samples (or below 3 reads = 10% × 30 reads). For the infant, the background consisted of all mothers (including mothers that were used to simulate the infant), real infant samples (excluding infants of mothers used to simulate the infant), and 337 samples of adults from the USA in the HMP (which includes 180 unique adults) that were obtained from the metagenomics repository of HMP under project ID SRP002163 and SRP056641 [20, 43]. For the mother, the background consisted of all mother and infant samples in addition to the HMP samples. For computing shared marker alleles, an allele must be present in both the mother and infant but absent from the background, which consisted of all mothers and the HMP samples.

To compute sharing, two quantities were considered: “total sharing,” defined as % shared marker alleles/ (infant marker alleles + mother marker alleles + shared marker alleles) and proportion of infant marker alleles that are shared: % shared marker alleles/ (infant marker alleles + shared marker alleles). The first quantity compared to FEAST estimates was the percentage of infant species in which the “total sharing” was at least 5%. The second quantity compared to FEAST was the pooled proportion of infant marker alleles that are shared across all species.

## Supplementary Information

**Additional file 1.** Supplementary materials. Contains Table S1, Figures S1-S8.

**Additional file 2.** Review history.

## Data Availability

SNV-FEAST signature SNV selection is implemented in Python and available for pip installation via https://pypi.org/project/Signature-SNVs [48]. Its source code, as well as analyses in this paper, is available at https://github.com/garudlab/Signature-SNVs [49], freely licensed under GPL3. The version used in this manuscript is permanently available at.https://doi.org/10.5281/zenodo.7515044 [50]. All metagenomic data was obtained from public repositories. The applicable accessions numbers are PRJEB6456 for Bäckhed et al. (mother-infant) [11], PRJEB323631 for Brooks et al. (NICU) [51], PRJEB402 for Sunagawa et al. (Tara Oceans) [52], and SRP002163 and SRP056641 for HMP [20, 43].

## References

[1] Knights D, Kuczynski J, Charlson ES, Zaneveld J, Mozer MC, Collman RG (2011). Bayesian community-wide culture-independent microbial source tracking. Nat Methods..

[2] Shenhav L, Thompson M, Joseph TA, Briscoe L, Furman O, Bogumil D (2019). FEAST: fast expectation-maximization for microbial source tracking. Nat Methods.

[3] Nayfach S, Rodriguez-Mueller B, Garud N, Pollard KS (2016). An integrated metagenomics pipeline for strain profiling reveals novel patterns of bacterial transmission and biogeography. Genome Res.

[4] Asnicar F, Manara S, Zolfo M, Truong DT, Scholz M, Armanini F, et al. Studying vertical microbiome transmission from mothers to infants by strain-level metagenomic profiling. mSystems. 2017;2(1). Available from: https://journals.asm.org/journal/msystems. [Cited 2021 Jun 14] 10.1128/mSystems.00164-16 PMC5264247 28144631

[5] Ferretti P, Pasolli E, Tett A, Asnicar F, Gorfer V, Fedi S (2018). Mother-to-infant microbial transmission from different body sites shapes the developing infant gut microbiome. Cell Host Microbe.

[6] Olm MR, Crits-Christoph A, Bouma-Gregson K, Firek BA, Morowitz MJ, Banfield JF (2021). inStrain profiles population microdiversity from metagenomic data and sensitively detects shared microbial strains. Nat Biotechnol 2021 396.

[7] Korpela K, Costea P, Coelho LP, Kandels-Lewis S, Willemsen G, Boomsma DI (2018). Selective maternal seeding and environment shape the human gut microbiome. Genome Res.

[8] Li SS, Zhu A, Benes V, Costea PI, Hercog R, Hildebrand F (2016). Durable coexistence of donor and recipient strains after fecal microbiota transplantation. Science (80-).

[9] Schmidt TSB, Hayward MR, Coelho LP, Li SS, Costea PI, Voigt AY (2019). Extensive transmission of microbes along the gastrointestinal tract. Elife.

[10] Schloissnig S, Arumugam M, Sunagawa S, Mitreva M, Tap J, Zhu A (2013). Genomic variation landscape of the human gut microbiome. Nature..

[11] Bäckhed F, Roswall J, Peng Y, Feng Q, Jia H, Kovatcheva-Datchary P (2015). Dynamics and stabilization of the human gut microbiome during the first year of life. Cell Host Microbe.

[12] Yassour M, Jason E, Hogstrom LJ, Arthur TD, Tripathi S, Siljander H (2018). Strain-level analysis of mother-to-child bacterial transmission during the first few months of life. Cell Host Microbe.

[13] Asnicar F, Manara S, Zolfo M, Truong DT, Scholz M, Armanini F, et al. Studying vertical microbiome transmission from mothers to infants by strain-level metagenomic profiling. mSystems. 2017;2(1). Available from: https://journals.asm.org/doi/abs/10.1128/mSystems.00164-16. [Cited 2022 Mar 7] 10.1128/mSystems.00164-16 PMC5264247 28144631

[14] Sprockett DD, Martin M, Costello EK, Burns AR, Holmes SP, Gurven MD (2020). Microbiota assembly, structure, and dynamics among Tsimane horticulturalists of the Bolivian Amazon. Nat Commun 2020 111.

[15] Sloan WT, Lunn M, Woodcock S, Head IM, Nee S, Curtis TP (2006). Quantifying the roles of immigration and chance in shaping prokaryote community structure. Environ Microbiol..

[16] Sloan WT, Woodcock S, Lunn M, Head IM, Curtis TP (2007). Modeling taxa-abundance distributions in microbial communities using environmental sequence data. Microb Ecol.

[17] Chen EZ, Li H (2016). A two-part mixed-effects model for analyzing longitudinal microbiome compositional data. Bioinformatics.

[18] Martin BD, Witten D, Willis AD (2020). Modeling microbial abundances and dysbiosis with beta-binomial regression. Ann Appl Stat..

[19] Consortium THM. Human Microbiome Project. 2013. Available from: https://aws.amazon.com/datasets/human-microbiome-project/.

[20] Lloyd-Price J, Mahurkar A, Rahnavard G, Crabtree J, Orvis J, Hall AB (2017). Strains, functions and dynamics in the expanded Human Microbiome Project. Nat..

[21] Brooks B, Olm MR, Firek BA, Baker R, Thomas BC, Morowitz MJ (2017). Strain-resolved analysis of hospital rooms and infants reveals overlap between the human and room microbiome. Nat Commun..

[22] Sunagawa S, Coelho LP, Chaffron S, Kultima JR, Labadie K, Salazar G, et al. Structure and function of the global ocean microbiome. Science (80- ). 2015;348(6237). Available from: https://science.sciencemag.org/content/348/6237/1261359. [Cited 2021 Jul 27] 10.1126/science.1261359 25999513

[23] Ladau J, Sharpton TJ, Finucane MM, Jospin G, Kembel SW, O’Dwyer J (2013). Global marine bacterial diversity peaks at high latitudes in winter. ISME J.

[24] Cavalli-Sforza LL, Feldman MW (2003). The application of molecular genetic approaches to the study of human evolution. Nat Genet.

[25] DeGiorgio M, Rosenberg NA (2013). Geographic sampling scheme as a determinant of the major axis of genetic variation in principal components analysis. Mol Biol Evol..

[26] Golani D. Distribution of Lessepsian migrant fish in the Mediterranean. 101080/11250009809386801. 2009;65(S1):95–9. Available from: https://www.tandfonline.com/doi/abs/10.1080/11250009809386801. [Cited 2022 Mar 6]

[27] Bentur Y, Ashkar J, Lurie Y, Levy Y, Azzam ZS, Litmanovich M (2008). Lessepsian migration and tetrodotoxin poisoning due to Lagocephalus sceleratus in the eastern Mediterranean. Toxicon.

[28] Bianchi CN, Morri C. Global sea warming and “tropicalization” of the Mediterranean Sea: biogeographic and ecological aspects. Biogeogr – J Integr Biogeogr. 2003;24(1). Available from: https://escholarship.org/uc/item/7bj69490. [Cited 2022 Mar 6]

[29] Elsaeed E, Fahmy N, Hanora A, Enany S (2021). Bacterial taxa migrating from the Mediterranean Sea into the Red Sea revealed a higher prevalence of anti-Lessepsian migrations. Omi A J Integr Biol.

[30] Antunes A, Ngugi DK, Stingl U (2011). Microbiology of the Red Sea (and other) deep-sea anoxic brine lakes. Environ Microbiol Rep.

[31] Flores GE, Bates ST, Knights D, Lauber CL, Stombaugh J, Knight R (2011). Microbial biogeography of public restroom surfaces. PLoS One..

[32] McGhee JJ, Rawson N, Bailey BA, Fernandez-Guerra A, Sisk-Hackworth L, Kelley ST (2020). Meta-SourceTracker: application of Bayesian source tracking to shotgun metagenomics. PeerJ.

[33] Austin GI, Park H, Meydan Y, Seeram D, Sezin T, Lou YC (2023). Contamination source modeling with SCRuB improves cancer phenotype prediction from microbiome data. Nat Biotechnol..

[34] Dlugosch L, Poehlein A, Wemheuer B, Pfeiffer B, Badewien TH, Daniel R (2022). Significance of gene variants for the functional biogeography of the near-surface Atlantic Ocean microbiome. Nat Commun..

[35] Delmont TO, Kiefl E, Kilinc O, Esen OC, Uysal I, Rappé MS (2019). Single-amino acid variants reveal evolutionary processes that shape the biogeography of a global SAR11 subclade. Elife.

[36] Alexander DH, Novembre J, Lange K (2009). Fast model-based estimation of ancestry in unrelated individuals. Genome Res..

[37] Chiu AM, Molloy EK, Tan Z, Talwalkar A, Sankararaman S. Inferring population structure in biobank-scale genomic data. Am J Hum Genet. 2022; Available from: https://linkinghub.elsevier.com/retrieve/pii/S0002929722000660. [Cited 2022 Mar 21] 10.1016/j.ajhg.2022.02.015 PMC9069078 35298920

[38] Siranosian BA, Tamburini FB, Sherlock G, Bhatt AS (2020). Acquisition, transmission and strain diversity of human gut-colonizing crAss-like phages. Nat Commun..

[39] Vatanen T, Jabbar KS, Vlamakis H, Knip M, Correspondence RJX (2022). Mobile genetic elements from the maternal microbiome shape infant gut microbial assembly and metabolism. Cell.

[40] Chen DW, Garud NR (2022). Rapid evolution and strain turnover in the infant gut microbiome. Genome Res.

[41] Katsanevakis S, Coll M, Piroddi C, Steenbeek J, Lasram FBR, Zenetos A (2014). Invading the Mediterranean Sea: Biodiversity patterns shaped by human activities. Front Mar Sci.

[42] Shi ZJ, Dimitrov B, Zhao C, Nayfach S, Pollard KS (2021). Fast and accurate metagenotyping of the human gut microbiome with GT-Pro. Nat Biotechnol..

[43] Consortium THM (2012). A framework for human microbiome research. Nat..

[44] Garud NR, Good BH, Hallatschek O, Pollard KS (2019). Evolutionary dynamics of bacteria in the gut microbiome within and across hosts. PLoS Biol..

[45] Langmead B, Salzberg SL (2012). Fast gapped-read alignment with Bowtie 2. Nat Methods..

[46] Hijmans RJ, Karney C, Geographiclib ] (, Williams E, Vennes C, Maintainer ]. Package “geosphere.” 2021;

[47] CRAN - Package ggpubr. Available from: https://cran.r-project.org/web/packages/ggpubr/index.html. [Cited 2022 Mar 6]

[48] Briscoe, Leah; Halperin, Eran; Garud N. Signature-SNVs. PyPi. 2023; Available from: https://pypi.org/project/Signature-SNVs/

[49] Briscoe, Leah; Halperin, Eran; Garud N. Signature-SNVs. Github. 2023. Available from: https://github.com/garudlab/Signature-SNVs

[50] Briscoe, Leah; Halperin, Eran; Garud N. Signature-SNVs. Zenodo. 2023. 10.5281/zenodo.7515044

[51] Brooks B, Firek BA, Miller CS, Sharon I, Thomas BC, Baker R (2014). Microbes in the neonatal intensive care unit resemble those found in the gut of premature infants. Microbiome..

[52] Zeller G, Tap J, Voigt AY, Sunagawa S, Kultima JR, Costea PI (2014). Potential of fecal microbiota for early-stage detection of colorectal cancer. Mol Syst Biol..

