# Supplementary material for: SNV-FEAST: microbial source tracking with single nucleotide variants
Source: Genome Biol. 2023 Apr 30;24:101. doi: 10.1186/s13059-023-02927-8 (PMC10150486; doi:10.1186/s13059-023-02927-8)
Supplement: Supplementary file 2 — Additional file 2. Review history. [file 13059_2023_2927_MOESM2_ESM.docx]

Review History

**First round of review**

**Reviewer 1**

**Were you able to assess all statistics in the manuscript, including the appropriateness of statistical tests used?** Yes, and I have assessed the statistics in my report.

**Comments to author:**

The manuscript from Briscoe and colleagues describes the application of an algorithm called 'FEAST' on single nucleotide variants from metagenomic samples of microbiomes. For a given microbiome, and a set of candidate 'source' microbiomes, FEAST claims to determine the proportional contributions of each source microbiome to seeding the recipient microbiome. While the original algorithm as published attempted to do this using species composition, this manuscript describes the novel application of it to single nucleotide variant (SNV) frequencies. The reasoning behind this is that a recipient microbiome will share strains with specific SNVs with a source microbiome.

On the whole, I think the authors have chosen reasonable datasets for re-analysis. I think that the mathematical and computational principles behind their algorithm are likely sound, and the writing is clear, as is the general logic. However, I find some greater conceptual issues and benchmarks that are missing from the work in its current form. Some of these are more general conceptual issues and some are more specific. I'd sympathize strongly with the feeling that general conceptual issues can be challenging to respond to, and because of this I'd feel like the authors could best respond to them by narrowing the scope of their claims and discussing the specific 'summarized' points directly below.

I apologize my review is so long. I realize a long review may be daunting to the authors, and that is not my intention. So first I will summarize my main points below for how to improve the manuscript:

1. Caveat the meaning of the word "source" and discuss the epistemological caveats therein.

2. Compare to existing methodology (popANI, MIDAS strain sharing) on each of the three datasets.

3. Provide clarity on the meaning of SNV sources in their results in a taxonomic context. (see below point '0b')

4. Show what happens when you exchange a given recipient with its sources, and re-run FEAST, on each of the 3 datasets. What do the results of this test mean, and what are the implications for interpretation?

General:

In general, I am *highly* skeptical of several greater principles at play here. I am skeptical that FEAST-species is capable of proving that recent transmission has directly occurred between two sampled microbiomes - let alone the directionality of that transmission. In essence, it seems to just report that a particular sample shares some unique or interesting species with another sample at particular frequencies. But this happens for many reasons, and does not indicate transmission. For example, if you passed FEAST a sample from antarctic soils, and asked it to quantify how much it was 'sourced' from a set of samples of (a) siberian soils, (b) human microbiomes, and (c) hot thermal springs, I suspect it would say that it was seeded by the siberian soils. But almst certainly the microbes in these physical locations were each seeded by elsewhere and have never interacted!

With SNVs, I believe that FEAST or similar approaches *may in principle* get at transmission. This is because private SNVs can represent recent evolutionary events. Essentially the transmission question of microbiomes is one of recency - on a long enough time scale, all microbiomes share a twisted and unknowable series of original "sources" - microbial dispersal has been ongoing since the dawn of life. When we ask "what is the source?" of a microbiome, we are actually asking what are the most recent or direct sources of microbes in that microbiome, which SNVs can represent.

However, still with SNVs, I am skeptical of FEAST because it does not seem to get at the directionality of transmission: instead my understanding is it seems to essentially report a similarity. Directionality of transmission is truly difficult, and might only be attempted with careful phylogenetics (although https://www.nature.com/articles/s41467-019-14103-3 this work attempts it in an interesting way, see Fig 2). To get at the transmission problem, and to convince me and readers, a step the authors could take is to demonstrate what happens when you flip a given source and the recipient in a FEAST analysis? Do the results change dramatically? If not, that would indicate it is merely reporting a form of similarity or overlap, not true transmission, and claims of "source" in the manuscript might have to be drastically toned down.

Maybe the above is obvious to the authors, and they would argue that this is a straightforward caveat, I am not sure. But I do genuinely think that many readers will take the usage of the term "source" at face-value, and genuinely believe that these methods can conclusively prove that a microbiome was seeded by a particular source. In general, my hope is that the authors could provide a strongly worded and thoughtful discussion of the caveats here and in general caveat their usage of the word "source" throughout the manuscript. But my worry is that an uncritical presentation of methods like FEAST as truly indicating evidence of microbial sources could lead to rampant misuse by readers (as is not uncommon with other tools in our field, sadly).

Specifics:

0. Most importantly, I think the results of SNV-FEAST need to be both (a) directly compared to existing methodology for strain transmissiong and (b) need to be explored in a taxonomic context. Both would greatly improve the clarity of this work.

(0a) Similar methodologies for detecting transmission events at the strain level have been developed by Nayfach 2016 and Olm 2021. The latter of which, popANI, may be especially insightful. PopANI essentially asks "how many unique alleles are there between two [per-species] populations?" The reasoning that popANI of 100% implies a transmission event is that for population A's allele to be a *perfect* subset of population B's alleles means they would have had to diverge relatively recently in evolutionary time, on the order of days to months.

(0b) Past works have analyzed strain transmission by comparing on the per strain level. This work groups all SNVs together, and indeed throughout there is no consideration of which species the SNVs belong to! However, this is very important to consider, both from a biological standpoint (it is interesting to know which species SNVs associate with a transmission event) as well as a technical standpoint. You can imagine doing permutations of SNV-FEAST where you withhold a random half of the species, or half of the SNVs from each species, recalculate sources, and then permute - this could help understand how robust a particular association is - does it depend on just a few SNVs, or just a few species?

1. Page 7, Line 14 "A single unknown source (e.g. a randomly selected unrelated mother) was also selected to contribute to the simulated infan"

I think that the simulations could be broadly improved by adding to the question of unknown sources. What happens when the proportion from unknown increases? How do samples that are 90% sourced from unknowns compare to 50%, 25%, 10%, etc? What does this mean for the real world, where in essence we do not know the % that any given sample may have been seeded from unsampled sources?

2. "We find that species-FEAST estimates an increasing contribution of unrelated mothers over time (p= 0.014) while SNV-FEAST estimates no significant change over time (p = 0.59)"

Does this quote not belie a serious issue with species-FEAST? I think it is straight-forwardly obvious what is happening in this case: the infant microbiomes are maturing into adult microbiomes. They almost certainly aren't being directly seeded by the *other* mothers in the study - rather they are simply obtainining microbes that are characteristic of adult microbiomes. But many readers who are not aware of the technicalities here, might read the above sentence and conclude that the infants microbiomes were seeded by the other mothers in the study! But I do not believe that is how we can interpret the above data, but am happy for the authors to describe why I might be wrong there.

3. "Interestingly, species-FEAST estimates a sharp decline in contribution of unknown sources (p=7.1 x 10-12) (Figure 2), whereas SNV-FEAST estimates little change in the contribution of unknown (p = 0.49) (Figure 2)."

Again, this finding raises a few serious points. First of all, what does it mean when species-FEAST and SNV-FEAST differ? In effect, determining the source of a microbiome at the species level or at the strain level should be equivalent - strains just make up species, after all. Whenever they differ, it means one approach has failed from a purely technical perspective!

Secondly, the observed ternd is the exact opposite of what we should expect! Early in life, it is generally observed that a large percentage of strains are inherited from the mother - and fewer later in life. The "% from unknown" should be increasing as the child encounters new and richer microbial environments - but here we see it decreasing at the species level and flatlining at the SNV level. While at the SNV level, the % unknown should increase drastically.

4. Compare Figure 2B - "% from mother" to Figure 3D from Nayfach et al. - at 0 days after birth, the mean of marker alleles shared is much higher in Nayfach than in this work. Nayfach implies that most of the strains found at Day 0 are from the mother, while this work implies a minority are - this is quite a discrepancy! Can the authors explain it further?

5. In the analysis of Brooks et al., it is difficult for me to intuit if there is a same-room signal. I do see four infants that are dominated by same room sources, but maybe 3 that aren't - can the authors summarize this data in a different way and perhaps justify it with statistical tests? If they could show that SNV-FEAST consistently predicts that an infant's microbiome was sourced more from its room than from others in this data, more than expected by chance, that would be convincing.

6. It is unclear to me if there are any significant trends presented in the ocean work. The correlations almost entirely seem statistically insignificant, and otherwise visually unimpressive - is there something I'm missing here. Further, I think it's a great case for the authors to grapple with the idea of "source". Consider comparing to Delmont et al, who concluded:

https://elifesciences.org/articles/46497

"Overall, these findings suggest that environmentally-mediated selection plays a critical role in the journey of cosmopolitan microbial populations in the surface ocean, lending credence to the idea for marine systems that 'everything is everywhere but the environment selects' (Baas-Becking, 1934).""

Thus, their conclusions run contrary to the assumption that geographically proximal sites will have similar microbiomes because geographic proximity lends itself to dispersal (as the authors assume) - rather, they are concluding that dispersal is nearly unlimited, and it is environmental selection that shapes the ocean microbiome. This easily could create an effect that appears like strain sourcing via geographic proximity as geographically proximal locations share similar environmental factors. This would inherently distort interpretation of strain sourcing - how would the authors respond?

7. The authors write: "However, this approach may miss strain sharing of lower-abundance strains whose haplotypes cannot be confidently resolved"

However, popANI (Olm et al. 2021) was devised entirely to also incorporate the abundances of lower-abundance alleles, and thus a comparison to popANI is highly warranted.

8. At the end, the authors write: "Source tracking provides insight into source contributions to a metagenomic sample as well as similarities between metagenomic samples"

An excellent summary of my main general thoughts is it is unclear to me that the methodology does indeed provide any insight into the former instead of just merely the latter.

**Reviewer 2**

**Were you able to assess all statistics in the manuscript, including the appropriateness of statistical tests used?** Yes, and I have assessed the statistics in my report.

**Comments to author:**

Briscoe et al. applied previous well-established MIDAS and FEAST workflow and analyzed microbiome source tracking based on SNVs. The idea is interesting, but the story overall is lack of novelty. I think many phrases can be reworded throughout the text to be simpler, clearer, and more direct. Besides, the way presented in several places were not clear to me. My major comments/suggestions are:

1)	Overall, the introduction is too long and not well organized. For example, the 3rd paragraph started with 16S data, which is not related the main information of the manuscript. Likewise, 4th paragraph listed bunch of references, readers easily loss of their interests. Currently, it seems mixture of both, which make less clear on the method strength itself. I would structure the introduction into a more method based-paper, and apple to real-world dataset for biology.

2)	The first part of results section stated from FEAST algorithm that only stated the background of the story, not providing any new information, which is disappointing.

3)	Several places in results section on the biological interpretation were not clear. For example, the text corresponding to figure 2 doesn’t provide too much biological interpretations.

4)	The method on the determining the signature SNV set is somewhat novel in the current method. However, this part is entirely missing in the results section and disappointing readers. I would emphasize this part in the result section.

5)	What the current outcome of the workflow is unclear to me. i.e. How does the current workflow adapt to the FEAST is not clear to me. or do authors want to present this as an advanced method of FEAST?

**Authors Response**

**Point-by-point responses to the reviewers’ comments:**

**Reviewer #1:** The manuscript from Briscoe and colleagues describes the application of an algorithm called 'FEAST' on single nucleotide variants from metagenomic samples of microbiomes. For a given microbiome, and a set of candidate 'source' microbiomes, FEAST claims to determine the proportional contributions of each source microbiome to seeding the recipient microbiome. While the original algorithm as published attempted to do this using species composition, this manuscript describes the novel application of it to single nucleotide variant (SNV) frequencies. The reasoning behind this is that a recipient microbiome will share strains with specific SNVs with a source microbiome.

On the whole, I think the authors have chosen reasonable datasets for re-analysis. I think that the mathematical and computational principles behind their algorithm are likely sound, and the writing is clear, as is the general logic. However, I find some greater conceptual issues and benchmarks that are missing from the work in its current form. Some of these are more general conceptual issues and some are more specific. I'd sympathize strongly with the feeling that general conceptual issues can be challenging to respond to, and because of this I'd feel like the authors could best respond to them by narrowing the scope of their claims and discussing the specific 'summarized' points directly below.

I apologize my review is so long. I realize a long review may be daunting to the authors, and that is not my intention. So first I will summarize my main points below for how to improve the manuscript:

We thank the reviewer for their kind words as well as their critical feedback which helped us to significantly improve the manuscript. Below we briefly summarize our response to points 1-4 and expand further within the response to reviews.

1. Caveat the meaning of the word "source" and discuss the epistemological caveats therein.

We thank the reviewer for pointing out this issue and have modified the introduction’s opening paragraph as well as discussion to discuss caveats to source tracking (see below for elaboration).

2. Compare to existing methodology (popANI, MIDAS strain sharing) on each of the three datasets.

We thank the reviewer for this suggestion and have made comparisons between SNV-FEAST and both inStrain’s popANI and the strain sharing approach in Nayfach 2016 (the MIDAS paper) (see below for elaboration).

3. Provide clarity on the meaning of SNV sources in their results in a taxonomic context. (see below point '0b')

We thank the reviewer for suggesting this and have performed experiments in which we withhold 10 and 50% of species or 10 and 50% of SNVs included for source tracking (see below for elaboration).

4. Show what happens when you exchange a given recipient with its sources, and re-run FEAST, on each of the 3 datasets. What do the results of this test mean, and what are the implications for interpretation?

We have performed ‘flipping experiments’ in which source and sink are swapped for each of the three case studies. We find that in all cases, the contributions from one sample to another (e.g. mother to infant) are asymmetric. However, we caveat strongly in the discussion that asymmetric contributions may not be conclusive evidence for directionality, especially in the case of ocean samples where transmissions may have occurred in the distant past.

General:

In general, I am *highly* skeptical of several greater principles at play here. I am skeptical that FEAST-species is capable of proving that recent transmission has directly occurred between two sampled microbiomes - let alone the directionality of that transmission. In essence, it seems to just report that a particular sample shares some unique or interesting species with another sample at particular frequencies. But this happens for many reasons, and does not indicate transmission. For example, if you passed FEAST a sample from antarctic soils, and asked it to quantify how much it was 'sourced' from a set of samples of (a) siberian soils, (b) human microbiomes, and (c) hot thermal springs, I suspect it would say that it was seeded by the siberian soils. But almst certainly the microbes in these physical locations were each seeded by elsewhere and have never interacted!

With SNVs, I believe that FEAST or similar approaches *may in principle* get at transmission. This is because private SNVs can represent recent evolutionary events. Essentially the transmission question of microbiomes is one of recency - on a long enough time scale, all microbiomes share a twisted and unknowable series of original "sources" - microbial dispersal has been ongoing since the dawn of life. When we ask "what is the source?" of a microbiome, we are actually asking what are the most recent or direct sources of microbes in that microbiome, which SNVs can represent.

We sincerely thank the reviewer for their thoughtful points here and agree with them completely. Firstly, we wish to clarify that the language of “source tracking” was established by others previously (Knights et al., 2011; Shenhav et al., 2019). These authors assumed these caveats of the work. We now have substantially revised the paper to reflect these caveats as well. In the first paragraph of the introduction, we state:

“While source tracking was developed as a way to quantitatively characterize a sample based on a set of samples with known origin, in most studies, the true source of samples may never be collected. In these cases, source tracking approaches are useful in identifying similarities between microbiome samples even if they cannot be used to definitively identify the true source of origin.”

In the ocean case study of our manuscript, we state:

“In the source tracking context, rather than defining sharing as evidence of a transmission event (which is more likely in mother-infant data), estimated source contributions at best explain the extent to which a given ocean sample resembles other ocean samples. On one extreme, an ocean sample might be entirely explainable by a single ocean’s samples, and at the other extreme, an ocean sample might be explainable by multiple oceans at the same time. Another alternative is for an ocean sample to not be explainable by any of the provided sources, resulting in a high unknown component and potentially suggesting high endemism. These source tracking estimates could be indicative of the extent to which oceans mix or may be reflective of similar niches.”

In the Discussion, we write:

“Thus, source tracking in certain instances, such as the ocean microbiome, at best reflects the extent of similarity between samples and is less conclusive about directionality. ”

Additionally, throughout the paper, we now caveat the word ‘source’ with ‘potential source’, or, ‘putative’ source to indicate that these are inferences.

However, still with SNVs, I am skeptical of FEAST because it does not seem to get at the directionality of transmission: instead my understanding is it seems to essentially report a similarity. Directionality of transmission is truly difficult, and might only be attempted with careful phylogenetics (although <https://www.nature.com/articles/s41467-019-14103-3> this work attempts it in an interesting way, see Fig 2). To get at the transmission problem, and to convince me and readers, a step the authors could take is to demonstrate what happens when you flip a given source and the recipient in a FEAST analysis? Do the results change dramatically? If not, that would indicate it is merely reporting a form of similarity or overlap, not true transmission, and claims of "source" in the manuscript might have to be drastically toned down.

We thank the reviewer for this suggestion and have performed source tracking in all three case studies in which we swap the source and sink. We find indeed that the contribution from one metagenomic sample to another is asymmetric, and potentially indicative of transmission. However, we completely agree with the reviewer that directionality of transmission may be difficult to infer. We write the following in the Discussion section:

“We note that source tracking provides insights into similarities between microbiomes and potential transmissions, though the directionality is less conclusive. It is possible that increased contributions in one direction but not the other is suggestive of directionality of transmission. For example, in the case of the mother-infant data from Backhed et al. 2015, FEAST predicted higher contribution from mother to baby than vice versa. This is consistent with work done on crAss-like phage transmissions between mother and infant in the same dataset that showed evidence of directionality by tracking the accumulation of mutations over time that are private to the infant and absent from the mother (Siranosian et al., 2020). But in the case of the ocean, it is possible that over longer time periods, differences in relative contributions from one part of the world to another (e.g. Mediterranean to Red Sea) are more reflective of local selection pressures that permit certain species and genotypes (Delmont et al., 2019). Thus, source tracking in certain instances, such as the ocean microbiome, at best reflects the extent of similarity between samples and is less conclusive about directionality.”

Please note we have added a citation to the Siranosian et al., 2020 article and are appreciative of the suggestion.

Additionally, throughout the results section, we are careful to caveat that directionality may not be conclusively inferred with source tracking:

In the case of the mother-infant case study (Backhed et al.):

“Finally, we sought to understand the effect of swapping sink and source in the re-analysis of Backhed et al. 2015 data. In **Figure 3G and H**, the infant at birth is the potential source and mother is the sink. The estimated contribution from baby to mother is significantly smaller (species-FEAST: 11.9 difference, Wilcoxon rank sum test p-value = 0.013; SNV-FEAST: 16.0 difference, p-value = 2.2 x 10^-5^) compared to that of mother to baby. This trend may be suggestive, but is not conclusive, of directionality, whereby a less diverse source is seeded by a more diverse source.”

In the case of the NICU case study (Brooks et al.), we write:

“We then asked the question: is the infant more explained by the built environment rather than vice-versa, the built environment is more explained by the infant. We tested this by swapping the infant and each of the three built environment sources (**Figure 4C and D**). The estimated contribution of room to infant is significantly higher than the estimated contribution of infant to room, but this asymmetry is more pronounced with SNV-FEAST. SNV-FEAST showed significantly higher contribution of room to infant for two of the three surface types (floor and isolette top: Wilcoxon rank sum test p-value = 7.00x 10^-9^, touched surface: p-value = 0.0058, sink basin: p-value = 0.274) while species-FEAST found this to be true for one of the three surface types (floor and isolette top: Wilcoxon rank sum test p-value = 7.1x 10^-5^, touched surface: p-value = 0.968, sink basin: p-value = 0.998).”

In the case of the oceans case study (Sunagawa et al), we write:

“Finally, we investigated whether some oceans have higher estimated contributions to other oceans than vice versa, potentially indicative of the directionality of transmissions (though see Discussion). Specifically, we investigated the relationship between the Red Sea to the Mediterranean Sea (**Figure 6C and D**). Migration from the Red Sea to the Mediterranean, known as Lessepsian migration, is well-documented for not only microorganisms but also macroorganisms like fish (Bentur et al., 2008; Bianchi & Morri, 2003; Golani, 2009). Anti-Lessepsian migration (Red Sea to Mediterranean), on the other hand, has been primarily thought to be rare due to the Additionally, recent studies suggest that there is also evidence for anti-Lessepsian migration of bacteria (Mediterranean to Red Sea) may be more common than Lessepsian migration (Elsaeed et al., 2021). Research studies find that Mediterranean has brine pools that produce similar a similar environment to the Red Sea’s (Antunes et al., 2011), allowing for bacteria from the MS to potentially thrive in the RS.”

Maybe the above is obvious to the authors, and they would argue that this is a straightforward caveat, I am not sure. But I do genuinely think that many readers will take the usage of the term "source" at face-value, and genuinely believe that these methods can conclusively prove that a microbiome was seeded by a particular source. In general, my hope is that the authors could provide a strongly worded and thoughtful discussion of the caveats here and in general caveat their usage of the word "source" throughout the manuscript. But my worry is that an uncritical presentation of methods like FEAST as truly indicating evidence of microbial sources could lead to rampant misuse by readers (as is not uncommon with other tools in our field, sadly).

Thank you for this suggestion and we have incorporated caveats as described above.

Specifics:

0. Most importantly, I think the results of SNV-FEAST need to be both (a) directly compared to existing methodology for strain transmission and (b) need to be explored in a taxonomic context. Both would greatly improve the clarity of this work.

(0a) Similar methodologies for detecting transmission events at the strain level have been developed by Nayfach 2016 and Olm 2021. The latter of which, popANI, may be especially insightful. PopANI essentially asks "how many unique alleles are there between two [per-species] populations?" The reasoning that popANI of 100% implies a transmission event is that for population A's allele to be a *perfect* subset of population B's alleles means they would have had to diverge relatively recently in evolutionary time, on the order of days to months.

We thank the reviewer for this suggestion and have included a comparison of SNV-FEAST with inStrain’s popANI and the strain tracking approach developed in Nayfach et al. 2016 (the MIDAS paper). We are grateful for this suggestion, as it allowed us to clarify benefits in using SNV-FEAST compared to inStrain and the approach in Nayfach et al.2016.

We found that the comparison of SNV-FEAST with inStrain and Nayfach et al. 2016 is most informative if done with simulations in which the mixture proportions of the sources is known. Comparing the three methods on all the real datasets would result in three comparisons of estimates for a quantity for which we do not know the ground truth.

In the introduction, we now write:

“While tracking strain transmissions with SNVs has been highly successful in a number of studies (Asnicar et al., 2017a; Ferretti et al., 2018; Korpela et al., 2018; Li et al., 2016; Nayfach et al., 2016; Olm et al., 2021; Schmidt et al., 2019) current approaches to strain tracking are limited. These methods provide binary information by inferring whether or not a strain transmission has occurred per species but they do not shed light on the relative proportions of microbiomes that are similar. A specific example of this is inStrain (Olm et al., 2021) which computes a pairwise population-level average nucleotide identity (popANI) between two samples. If an infant harbors several strains derived from the mother at low frequency, these shared strains will have high popANI values, but they will represent a relatively small proportion of the infant’s microbiome. By contrast, source tracking allows us to simultaneously infer the putative proportions for multiple sources contributing to a given sink, integrated over all community members in the sink. As shown in **Figure 1**, one may be able to estimate that an infant microbiome is explained 25% by the mother, 10% by the dog, and 30% by unknown sources (Knights et al., 2011; Shenhav et al., 2019). In other words, source tracking with SNVs leverages not only the genetic variants within species, but also the relative abundances of the species that carry the SNVs.”

In the results section, we now write:

“To illustrate the advantage of SNV-FEAST over traditional strain tracking approaches such as inStrain (Olm et al., 2021), we used the same synthetic communities produced in the above simulation for inStrain profiling between each infant and each of their potential contributing sources (**Figure S2**). InStrain computes a popANI score, which represents the average nucleotide identity between two different metagenomic samples for a given species. As per the inStrain paper, popANI values > 99.999% represent the same strain for that species being shared between samples (**Methods**). However, this approach provides a binarization as to whether or not a strain was transmitted, and does not account for the relative abundance of the strain in the sink. Thus, we computed the fraction of each infant’s species that have popANI $\geq$99.999%, with each potential source.

As expected, both SNV-FEAST and inStrain produce estimates of sharing that correlate positively with the ground truth mixture proportions of the contributing source samples in each infant (**Figure S2**). We found inStrain results yielded a 0.742 Pearson correlation (p < 1x10^-12^) with the true mixture proportions, whereas SNV-FEAST has a 0.866 Pearson correlation (p < 1x 10^-12^) with the true proportions. The higher correlation values for SNV-FEAST likely reflect that relative abundances of strains and their genomic identities are simultaneously taken into account for source tracking, whereas inStrain only accounts for genomic identities. Finally, several of the shared species in the simulations had popANI values < 99.999%, reflecting the complex mixtures from multiple sources

We next compared SNV-FEAST with the strain tracking procedure in Nayfach et al. 2016. Again, we used the same synthetic communities produced in the simulation to determine marker alleles as defined in Nayfach et al. 2016 (**Methods**). Here a marker allele is determined to be a SNV that is private to mother, infant, or the mother-infant dyad, and absent from the background population, which consisted of other samples in the dataset as well as samples from United States adults in the Human Microbiome Project (**Methods**). Species with $\geq$5% marker allele sharing between mother and infant were deemed to share a strain (**Methods**). We found a high correlation between the true mixture proportions (on x-axis) and the percentage of species with transmission events (y-axis) (Pearson correlation 0.915 , p < 1 x 10^-16^). The higher correlation for the Nayfach et al. 2016 approach compared to the inStrain approach possibly reflects horizontal gene transfers between lineages residing in infants and mothers. By contrast, there was a lower correlation between the true mixture proportions (x-axis) and the sharing for all marker alleles across species present in the infant (y-axis) and (0.575 Pearson correlation, p < 1 x 10^-16^) (**Figure S3B**).”

(0b) Past works have analyzed strain transmission by comparing on the per strain level. This work groups all SNVs together, and indeed throughout there is no consideration of which species the SNVs belong to! However, this is very important to consider, both from a biological standpoint (it is interesting to know which species SNVs associate with a transmission event) as well as a technical standpoint. You can imagine doing permutations of SNV-FEAST where you withhold a random half of the species, or half of the SNVs from each species, recalculate sources, and then permute - this could help understand how robust a particular association is - does it depend on just a few SNVs, or just a few species?

We thank the reviewer for this suggestion. We have added these simulations to the manuscript and now write in the results section:

“To assess whether all species and all signatures SNVs in the sink are needed for accurate source tracking, we varied the proportion of species (from 10%, 50% or 100%) and SNVs (from 10%, 50% or 100%) included as inputs to the algorithm (**Figure S1**). We used Pearson correlation between the true and estimated proportions to represent accuracy of SNV-FEAST. When decreasing the percentage of SNVs used, there is no statistically significant change in the performance. However, when decreasing the percentage of species used, there are statistically significant decreases in the performance (**Figure S1**).”

Additionally, **Figure S3** (Figure S1 in original manuscript) shows the distribution of signature SNVs across species in the Backhed infant gut samples. We describe this figure in the Discussion:

“Another potential caveat of SNV filtering is that not all species present will be represented in the final signature SNV set (**Figure S4**). Species with higher abundance are more likely to be represented in the signature SNV set. However, we show that not all species need to contribute signature SNVs in order to make accurate inferences, and likewise, not all SNVs are needed to make accurate inferences (**Figure S1)**.”

1. Page 7, Line 14 "A single unknown source (e.g. a randomly selected unrelated mother) was also selected to contribute to the simulated infan"

I think that the simulations could be broadly improved by adding to the question of unknown sources. What happens when the proportion from unknown increases? How do samples that are 90% sourced from unknowns compare to 50%, 25%, 10%, etc? What does this mean for the real world, where in essence we do not know the % that any given sample may have been seeded from unsampled sources?

We thank the reviewer for this suggestion. We have now included proportions of unknown sources in our simulations ranging from 10% to 90% as described in Supplemental Table 1 (shown below). In **Figure 2**, we make explicit how well FEAST is able to recapitulate % unknown by showing the estimates of unknown distinctly in the graph. We find that SNV-FEAST outperforms species-FEAST, even where % unknown is as high as 90% and that the unknown component specifically is estimated more accurately by SNV-FEAST.

Supplemental Table 1

2. "We find that species-FEAST estimates an increasing contribution of unrelated mothers over time (p= 0.014) while SNV-FEAST estimates no significant change over time (p = 0.59)"

Does this quote not belie a serious issue with species-FEAST? I think it is straight-forwardly obvious what is happening in this case: the infant microbiomes are maturing into adult microbiomes. They almost certainly aren't being directly seeded by the *other* mothers in the study - rather they are simply obtainining microbes that are characteristic of adult microbiomes. But many readers who are not aware of the technicalities here, might read the above sentence and conclude that the infants microbiomes were seeded by the other mothers in the study! But I do not believe that is how we can interpret the above data, but am happy for the authors to describe why I might be wrong there.

We thank the reviewer for this question and have substantially rewritten this section of the results to make clear what the results mean. We now write in the results section:

“The increase in contribution from unrelated mothers with species-FEAST does not suggest that these particular unrelated mothers are seeding the infant. Rather, the opposing trend observed with SNVs suggests that similarity at the species level is consistent with the maturation of the infant microbiome over time”

3. "Interestingly, species-FEAST estimates a sharp decline in contribution of unknown sources (p=7.1 x 10-12) (Figure 2), whereas SNV-FEAST estimates little change in the contribution of unknown (p = 0.49) (Figure 2)."

Again, this finding raises a few serious points. First of all, what does it mean when species-FEAST and SNV-FEAST differ? In effect, determining the source of a microbiome at the species level or at the strain level should be equivalent - strains just make up species, after all. Whenever they differ, it means one approach has failed from a purely technical perspective!

Secondly, the observed ternd is the exact opposite of what we should expect! Early in life, it is generally observed that a large percentage of strains are inherited from the mother - and fewer later in life. The "% from unknown" should be increasing as the child encounters new and richer microbial environments - but here we see it decreasing at the species level and flatlining at the SNV level. While at the SNV level, the % unk nown should increase drastically.

In addressing this comment, we realized we did not clearly explain how source tracking was performed in the Backhed data. Our source tracking analysis included previous time points of the infant as potential sources, in addition to the mother, unrelated mothers, and unknown. By including previous time points of the infant, the interpretation of % unknown becomes clearer. We describe this in the re-written results:

“In applying FEAST to the Backhed dataset, we estimated the proportion of infant at birth attributable to mother. For 4 month infants, we estimated the proportion attributable to the mother and itself at birth. For 12 month infants, we estimated the proportion attributable to the mother and itself at birth and four months (Shenhav et al. 2019). This allowed “unknown” to be more strictly defined as the component of the infant microbiome that could not be explained by the mother. It also allowed us to better discern if completely new strains were acquired at the 4^th^ and 12^th^ months of life (that were not already acquired during previous life stages).”

We expect the overall unknown proportion to increase, though the way in which the results were presented previously suggested there was no change. We agree with the reviewer that we would expect the infant to encounter new diverse microbial environments overtime. In fact, when we consider the totality of both newly acquired microbes from unknown sources and previously acquired microbes from unknown sources, there is an overall increase. The results in **Figure 3** show the newly acquired microbes of unknown source, conditional on what was already present in an infant in a previous time point. **Figure S5** (not included in the previous manuscript) shows that the estimated unknown component is increasing over the first year of life when source tracking is run without the inclusion of previous time points as sources for SNVs only. We explain better what these results mean:

“Finally, we estimated contributions from unknown sources, i.e. the proportion of the infant microbiome not explainable by the true mother, the three randomly selected unrelated mothers, or any previous time point. Species-FEAST estimates a sharp decline in contribution of unknown sources over the first year of life (t-test p-value =7.1 x 10^-12^) (**Figure 3**). This significant decrease in unknown at the species level reflects the infant microbiome maturation over the first year of life. By contrast, SNV-FEAST estimates little change in the contribution of unknown sources (t-test p-value = 0.49) (**Figure 3**). Note that this unknown component reflects what was gained since a previous time point. In other words, at 12 months, the infant on average acquired the same fraction of unknown as it did at 4 months and birth. When source tracking is run without including previous time points as sources, the unknown component increases over the first year of life for SNVs only (**Figure S5)**.”

Additionally, to address the question of “what does it mean when species-FEAST and SNV-FEAST differ?” we have now added to our discussion the following:

“In application of species and SNV-FEAST to simulations as well as three case studies, we demonstrate that the two input types can provide distinct insights into microbial sharing and similarities across different environments. As a hypothetical example, two unrelated samples may have very similar species composition due to similar colonization processes and similar environmental influences without any actual microbial sharing. It would be unlikely for these two unrelated samples to share rare SNVs, however. This distinction suggests that SNVs indeed can provide insight into the ecological processes shaping microbial communities that species information alone cannot, and our three case studies are able to demonstrate this.”

An example of this meaningful distinction between SNV-FEAST and species-FEAST is excerpted below:

“Interestingly, the built environments of different rooms highly resemble each other. This is especially apparent with species-FEAST, suggestive of similar ecological forces operating in similar built environments. By contrast, SNV-FEAST reveals a higher diversity of contributing sources of the built environment samples to other NICU built environments, once again highlighting the utility of performing source tracking with SNVs.”

4. Compare Figure 2B - "% from mother" to Figure 3D from Nayfach et al. - at 0 days after birth, the mean of marker alleles shared is much higher in Nayfach than in this work. Nayfach implies that most of the strains found at Day 0 are from the mother, while this work implies a minority are - this is quite a discrepancy! Can the authors explain it further?

We thank the reviewer for pointing this out as this gives us the opportunity to distinguish between our approach and the approaches used in Nayfach et al. 2016 as well as inStrain. The major distinguishing feature is that our approach allows us to quantify compositionality as opposed to only a binarization as to whether or not a strain was transmitted. The approach of Nayfach et al. 2016 involves computing marker alleles and then quantifying the proportion of mother’s marker alleles that are shared with the infant. Using marker alleles as the denominator here restricts the analysis to only marker alleles. However there are portions of bacterial genomes that do not harbor marker alleles. We agree it’s surprising that the component explained by infant is low for mother. But this may be reflective of our use of all SNVs collectively irrespective of whether they are private to a mother-infant dyad.

In Figure S3 B, we directly compare the ability of SNV-FEAST and Nayfach et al’s marker alleles to recapitulate the true contribution of the mother to infant. We computed the percentage of infant marker alleles (shared with mother and private) that are shared with mother, and compared this to FEAST estimates of the percentage infant microbiome explained by mother. We found that SNV-FEAST more accurately estimated the true contribution of mother, and in general had higher estimates than the Nayfach et al. approach.

5. In the analysis of Brooks et al., it is difficult for me to intuit if there is a same-room signal. I do see four infants that are dominated by same room sources, but maybe 3 that aren't - can the authors summarize this data in a different way and perhaps justify it with statistical tests? If they could show that SNV-FEAST consistently predicts that an infant's microbiome was sourced more from its room than from others in this data, more than expected by chance, that would be convincing.

We thank the reviewer for this suggestion, and have presented the same data in a new way that makes more clear the trends of this analysis. This figure also includes the results of a Wilcoxon rank sum paired statistical test where the total contribution of samples from the built environment in the infant’s actual room are compared to the other room, per time point. We present the p-values of this test per infant. As shown in **Figure 4** replicated below, species-FEAST finds a larger contribution from the same room than other room for two of four infants, whereas SNV-FEAST finds this for three of four infants.

We now write in results:

“Through application of SNV and species-FEAST, we are able to quantify any trends over time the influence of the built environment on the infant microbiome (**Figures 4A and B**). SNV-FEAST more consistently finds that contribution from the infant’s own room exceeds contributions from a different room over time (paired Wilcoxon signed rank test for same room > different room: Infant 3: p-value = 1.95 x 10^-9^, Infant 6: 1, Infant 12: 3.05 x 10^-5^, Infant 18: 3.81 x 10^-6^) as compared to species-FEAST (Infant 3: p-value = 0.41, Infant 6: 1, Infant 12: 5.8 x 10^-4^, Infant 18: 3.81 x 10^-6^). Interestingly, species-FEAST assigns one dominant source primarily, whereas SNV-FEAST more often finds a combination of sources for a given sample.”

6. It is unclear to me if there are any significant trends presented in the ocean work. The correlations almost entirely seem statistically insignificant, and otherwise visually unimpressive - is there something I'm missing here.

We thank the reviewer for pointing this out and have redone our analyses with two major changes. First, our former distance decay work focused on only one ocean at a time, however we realized we could better detect general trends by including all oceans. We find a significant downward slope for both species and SNV-FEAST. We have replaced the previous figure with a new one, which is shown below along with the accompanying text:

“Next, we assessed whether source tracking estimates display a distance-decay relationship. Previous studies found that genetic distance, such as that represented by fixation index F_ST_, increases with geographic distance between populations (Cavalli-Sforza & Feldman, 2003; DeGiorgio & Rosenberg, 2013). Based on these findings, our expectation was that samples that are further away from a given station will have reduced resemblance to that station. To assess this distance-decay relationship, we plotted pairwise source tracking results across all possible pairs of ocean samples (**Figure 6A and B**). We found that indeed as the distance increases, the % explainability of a given source ocean decreases -0.23 % per thousand km according to species-FEAST (t-test p-value < 1 x 10^-16^), and -0.5% per thousand km according to SNV-FEAST (t-test p-value = 0.0018). The steeper slope for SNV-FEAST suggests that SNVs may be more sensitive to distance decay signals on a global level.”

**Figure 6A and B** are replicated below:

Second, we performed a “flipped analysis” in which samples from one ocean served as a sink and the other ocean’s samples a source, and vice versa. In this analysis we see statistically significantly different results in the relative contributions from one part of the ocean to another. Below we replicate **Figure 6C and D** where we show this.

Further, I think it's a great case for the authors to grapple with the idea of "source". Consider comparing to Delmont et al, who concluded:

<https://elifesciences.org/articles/46497>

"Overall, these findings suggest that environmentally-mediated selection plays a critical role in the journey of cosmopolitan microbial populations in the surface ocean, lending credence to the idea for marine systems that 'everything is everywhere but the environment selects' (Baas-Becking, 1934).""

Thus, their conclusions run contrary to the assumption that geographically proximal sites will have similar microbiomes because geographic proximity lends itself to dispersal (as the authors assume) - rather, they are concluding that dispersal is nearly unlimited, and it is environmental selection that shapes the ocean microbiome. This easily could create an effect that appears like strain sourcing via geographic proximity as geographically proximal locations share similar environmental factors. This would inherently distort interpretation of strain sourcing - how would the authors respond?

We thank the reviewer for pointing this out and completely agree with them. We have first toned down the language in the results section and also pointed to selection / shared environmental factors as being a reason for observing similarities between parts of the ocean.

Second, as described above, we have added a paragraph to the Discussion section caveating the meaning of source tracking and any inference of directionality especially in the context of the ocean analysis. We acknowledge that that selection and shared environmental factors could be a play. We further cite the Delmont article to illustrate this point (thank you for suggesting it).

7. The authors write: "However, this approach may miss strain sharing of lower-abundance strains whose haplotypes cannot be confidently resolved"

However, popANI (Olm et al. 2021) was devised entirely to also incorporate the abundances of lower-abundance alleles, and thus a comparison to popANI is highly warranted.

Thank you for this suggestion. As described above, we have included a comparison to popANI as well as the strain sharing approach outlined in Nayfach et al. 2016. We also have revised the language of the Discussion section to clarify that popANI does not rely on strain haplotype phasing and have deleted the passage about missing strain sharing of lower-abundance strains.

8. At the end, the authors write: "Source tracking provides insight into source contributions to a metagenomic sample as well as similarities between metagenomic samples"

An excellent summary of my main general thoughts is it is unclear to me that the methodology does indeed provide any insight into the former instead of just merely the latter.

Thank you to the reviewer for these comments. We hope the changes made throughout the manuscript better emphasize the caveats as well as benefits of source tracking in the microbiome.

**Reviewer #2:** Briscoe et al. applied previous well-established MIDAS and FEAST workflow and analyzed microbiome source tracking based on SNVs. The idea is interesting, but the story overall is lack of novelty. I think many phrases can be reworded throughout the text to be simpler, clearer, and more direct. Besides, the way presented in several places were not clear to me. My major comments/suggestions are:

1)	Overall, the introduction is too long and not well organized. For example, the 3rd paragraph started with 16S data, which is not related the main information of the manuscript. Likewise, 4th paragraph listed bunch of references, readers easily loss of their interests. Currently, it seems mixture of both, which make less clear on the method strength itself. I would structure the introduction into a more method based-paper, and apple to real-world dataset for biology.

We thank the reviewer for pointing out these areas for improvement and have implemented their suggestions as follows:

First, we cut the reference to 16S data in the 3rd paragraph and merge it with the second paragraph to more quickly make the point that species information lacks resolution about SNVs.

Second, we removed the numerous references to strain haplotype phasing in the 4th paragraph and instead more quickly address the limitations of strain-tracking with SNVs so that the benefit of source tracking becomes more apparent.

Third, we cut the paragraph on transmissions in the more distant past in non-human sources and have included discussion of this point in the Discussion section

Finally, we more pointedly explain in the final paragraph of the Introduction that we propose an extension of the FEAST algorithm so that it is applicable to SNVs. We point out that we benchmark the method in simulations and apply it to three real-world case studies. We have streamlined the final paragraph of the Introduction to reflect this.

All three of the main results are all from applications to real-world datasets for biology.

2)	The first part of results section stated from FEAST algorithm that only stated the background of the story, not providing any new information, which is disappointing.

We thank the reviewer for pointing this out. We have cut all background information from the first part of the result section (two paragraphs) and immediately delve into the details of the computational challenges pertaining to adapting FEAST to SNVs as input.

3)	Several places in results section on the biological interpretation were not clear. For example, the text corresponding to figure 2 doesn't provide too much biological interpretations.

We thank the reviewer for their suggestions and have added biological interpretations throughout the text. We now add sentences throughout that indicate the specific utility of our methods as well as the biological interpretation of our findings, as shown in the following excerpts:

For **Figure 3** (formerly Figure 2), we first provide the reader with an expectation based on previous work with this data:

“This dataset is composed of metagenomic samples from infants collected at four days, four months, and 12 months after birth, as well as their mothers at the time of delivery. Previous analyses on this data have shown even while species similarity increases, infants and their mothers share fewer proportions of strains over time as revealed by sharing of SNVs private to mother-infant dyads (Nayfach et al., 2016). Thus, SNVs belonging to strains shared only by the infant and their mother may be more informative of the true source compared to species. Here we sought to test whether SNV and species-FEAST recapitulate these results (**Methods**).”

We have completely rewritten this section to provide better biological context for our results:

“First, consistent with previous findings made with species and SNVs (Nayfach et al., 2016), species-FEAST estimates an increasing contribution of the mother over time (p = 5.1 x 10^-4^), but SNV-FEAST estimates a decrease over time (p = 0.063) (**Figure 3**).

Second, we assessed the ability of species and SNV-FEAST to distinguish the true mother from three randomly selected unrelated mothers. Species-FEAST estimates an increasing contribution of unrelated mothers over time (p= 0.014) while SNV-FEAST estimates no significant change over time (p = 0.59) (**Figure 3**). The increase in contribution from unrelated mothers with species-FEAST does not suggest that these particular unrelated mothers are seeding the infant. Rather, the opposing trend observed with SNVs suggests that similarity at the species level is consistent with the maturation of the infant microbiome over time.

Finally, we estimated contributions from unknown sources, i.e. the proportion of the infant microbiome not explainable by the true mother, the three randomly selected unrelated mothers, or any previous time point. Species-FEAST estimates a sharp decline in contribution of unknown sources over the first year of life (t-test p-value =7.1 x 10^-12^) (**Figure 3**). This significant decrease in unknown at the species level reflects the infant microbiome maturation over the first year of life. By contrast, SNV-FEAST estimates little change in the contribution of unknown sources (t-test p-value = 0.49) (**Figure 3**). Note that this unknown component reflects what was gained since a previous time point. In other words, at 12 months, the infant on average acquired the same fraction of unknown as it did at 4 months and birth. When source tracking is run without including previous time points as sources, the unknown component increases over the first year of life for SNVs only (**Figure S5)**.”

For the NICU data analysis, we improved interpretability by adding explanations and additional analyses:

“We applied SNV and species-FEAST to assess the contribution of the infant’s own NICU room as well as a different NICU room in the vicinity of the infant’s gut microbiome (**see Methods**)… We then asked the question: is the infant more explained by the built environment rather than vice-versa, the built environment is more explained by the infant. We tested this by swapping the infant and each of the three built environment sources (**Figure 4C and D**). The estimated contribution of room to infant is significantly higher than the estimated contribution of infant to room, but this asymmetry is more pronounced with SNV-FEAST. SNV-FEAST showed significantly higher contribution of room to infant for two of the three surface types (floor and isolette top: Wilcoxon rank sum test p-value = 7.00x 10^-9^, touched surface: p-value = 0.0058, sink basin: p-value = 0.274) while species-FEAST found this to be true for one of the three surface types (floor and isolette top: Wilcoxon rank sum test p-value = 7.1x 10^-5^, touched surface: p-value = 0.968, sink basin: p-value = 0.998). Interestingly, the built environments of different rooms highly resemble each other. This is especially apparent with species-FEAST, suggestive of similar ecological forces operating in similar built environments. By contrast, SNV-FEAST reveals a higher diversity of contributing sources of the built environment samples to other NICU built environments, once again highlighting the utility of performing source tracking with SNVs.”

For the oceans analysis, we provide more detailed biological context:

“We found that species and SNV-FEAST estimate different amounts of sharing between oceans, where SNVs estimate a higher unknown on average, potentially indicative of endemism. The finding that SNV-FEAST estimates a higher unknown contribution on average is most evident in the North Pacific, North Atlantic, South Atlantic, and Mediterranean oceans (**Figure S7**).”

“Next, we assessed whether source tracking estimates display a distance-decay relationship. Previous studies found that genetic distance, such as that represented by fixation index F_ST_, increases with geographic distance between populations (Cavalli-Sforza & Feldman, 2003; DeGiorgio & Rosenberg, 2013). Based on these findings, our expectation was that samples that are further away from a given station will have reduced resemblance to that station. To assess this distance-decay relationship, we plotted pairwiseh source tracking results across all possible pairs of ocean samples (**Figure 6A and B**). We found that indeed as the distance increases, the % explainability of a given source ocean decreases -0.23 % per thousand km according to species-FEAST (t-test p-value < 1 x 10^-16^), and -0.5% per thousand km according to SNV-FEAST (t-test p-value = 0.0018 ). The steeper slope for SNV-FEAST suggests that SNVs may be more sensitive to distance decay signals on a global level.”

4)	The method on the determining the signature SNV set is somewhat novel in the current method. However, this part is entirely missing in the results section and disappointing readers. I would emphasize this part in the result section.

We thank the reviewer for suggesting this. We have now added a dedicated section to the results describing the ‘signature score’ approach we develop. Additionally, we have included a schematic of our approach in **Figure 1**, shown below:

5)	What the current outcome of the workflow is unclear to me. i.e. How does the current workflow adapt to the FEAST is not clear to me. or do authors want to present this as an advanced method of FEAST?

We appreciate this comment as it helped us to clarify the contribution of work from the beginning of the manuscript. We have clarified the writing in several places to address this point.

Our abstract now includes the following:

“To overcome the computational burden of utilizing all SNVs for a given sample, we introduce a novel method to identify signature SNVs for source tracking. We show that signature SNVs used as input into a previously designed source tracking algorithm, FEAST, can more accurately estimate contributions than species and provide novel insights, demonstrated in three case studies.”

In the introduction, we explain the innovation of our manuscript is in creation of new input data and interpretation of results:

“Here, we evaluate whether source contributions estimated with SNVs are more accurate than with only species when provided as inputs to FEAST (Shenhav et al., 2019) (hereafter referred to as SNV-FEAST and species-FEAST, respectively). FEAST (Shenhav et al., 2019) is faster and more accurate than previous source tracking tools (Knights et al., 2011), and therefore, is ideal for adaptation to SNV source tracking since it can accept larger numbers of features and input sources. Despite this improved computational efficiency, the potentially millions of single nucleotide variants across all microbiome species in a given host still can computationally overwhelm FEAST. **To address this, we introduce a novel approach to determine signature SNVs that can be used as input to FEAST.** This both reduces memory requirements and computation time in the FEAST estimation, allowing us to optimally estimate the source contribution of a sink. We find that SNV-FEAST and species-FEAST yield different outcomes when applied to simulated data, with SNV-FEAST frequently out-performing species-FEAST”

Additionally, our schematic in **Figure 1** (shown above) clarifies the general outcome of the workflow.

**Second round of review**

**Reviewer 1**

The authors have responded thoroughly to my comments in their updated response, and the manuscript is significantly improved. They have added several additional benchmarks, expanded their discussion, and have included a new python package to identify Signature SNVs.

**Reviewer 2**

The authors have addressed all my questions.
